# Molecularly precise self-assembly of theranostic nanoprobes within a single-molecular framework for *in vivo* tracking of tumor-specific chemotherapy†
†Electronic supplementary information (ESI) available. See DOI: 10.1039/c8sc01069b


**DOI:** 10.1039/c8sc01069b

**Published:** 2018-04-24

**Authors:** Chenxu Yan, Zhiqian Guo, Yanyan Shen, Yi Chen, He Tian, Wei-Hong Zhu

**Affiliations:** a Key Laboratory for Advanced Materials , Institute of Fine Chemicals , Shanghai Key Laboratory of Functional Materials Chemistry , School of Chemistry and Molecular Engineering , East China University of Science and Technology , Shanghai 200237 , China . Email: whzhu@ecust.edu.cn ; Email: guozq@ecust.edu.cn; b Division of Anti-Tumor Pharmacology , State Key Laboratory of Drug Research , Shanghai Institute of Materia Medica , Chinese Academy of Sciences , Shanghai 201203 , China

## Abstract

Structural heterogeneity and the lack of *in vivo* real-time tracking of drug release are the utmost barriers for nanocarrier-mediated prodrugs in targeted therapy. Herein, we describe the strategy of molecularly precise self-assembly of monodisperse nanotheranostics for BP_*n*_-DCM-S-CPT (*n* = 0, 5 and 20) with fixed drug loadings (36%, 23% and 16%) and constant release capacities, permitting *in vivo* real-time targeted therapy. We focus on regulating the hydrophilic fragment length to construct stable, well-defined nanostructured assemblies. Taking the bis-condensed dicyanomethylene-4*H*-pyran (DCM) derivative as the activatable near-infrared (NIR) fluorophore, it makes full use of two terminal conjunctions: the hydrophobic disulfide-bridged anticancer prodrug camptothecin (CPT) and the hydrophilic oligomer-bridged biotin segment serving as an active targeting unit. From the rational design, only BP_20_-DCM-S-CPT forms uniform and highly stable self-assemblies (*ca.* 80 nm, critical micelle concentration = 1.52 μM) with several advantages, such as structural homogeneity, fixed drug loading efficiency, real-time drug release tracking and synergistic targeting (passive, active and activatable ability). More importantly, *in vitro* and *in vivo* experiments verify that the surface-grafted biotins of nanoassemblies are directly exposed to receptors on cancer cells, thus markedly facilitating cellular internalization. Notably, through synergistic targeting, BP_20_-DCM-S-CPT displays excellent tumor-specific drug release performance in HeLa tumor-bearing nude mice, which has significantly enhanced *in vivo* antitumor activity and nearly eradicates the tumor (IRT = 99.7%) with few side effects. For the first time, the specific molecularly precise self-assembly of BP_20_-DCM-S-CPT within a single-molecular framework has successfully achieved a single reproducible entity for real-time reporting of drug release and cancer therapeutic efficacy in living animals, providing a new insight into amphiphilic nanotheranostics for clinical translation.

## Abstract 2

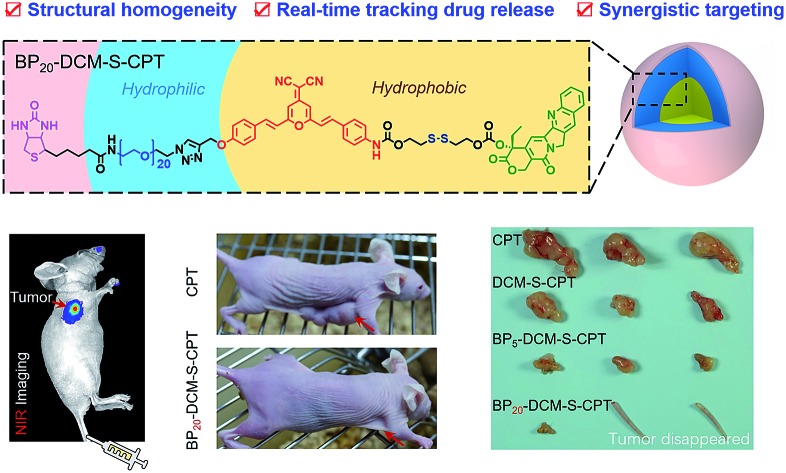
The strategy of molecularly precise self-assembly of theranostic nanoprobes within a single-molecular framework is used to avoid batch-to-batch variability, and concurrently achieving real-time tracking of the *in vivo* behaviour of prodrugs for the first time.

## Introduction

**Fig. 1:**
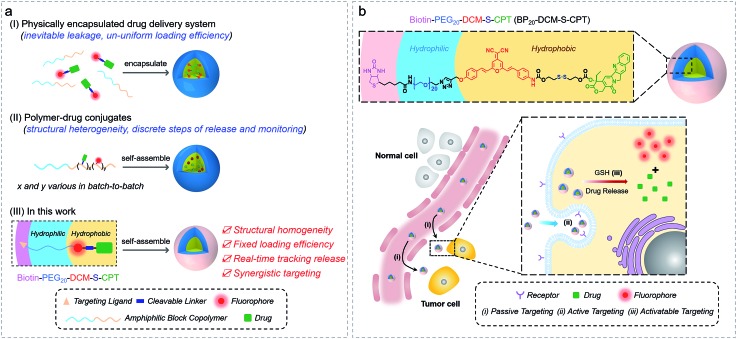
Amphiphilic self-assembled nanotheranostic core–shell systems. (a) Schematic illustration of general prodrug loading strategies: (I) physically encapsulated drug delivery system, physical encapsulation of the drug into the amphiphilic block copolymer, (II) polymer–drug conjugates, grafting the drug onto the amphiphilic polymer chain *via* covalent bonding, and (III) molecularly precise self-assembled amphiphilic theranostics, integrating the advantageous features of small molecular theranostics and polymer–drug conjugates. (b) Tumor specific molecularly precise self-assembled nanotheranostics for BP_20_-DCM-S-CPT with enhanced synergistic targeting including (i) passive targeting from the preferable micelle-based EPR effect, (ii) active targeting from shell surface-grafted biotin directly exposed to receptors on cancer cells for markedly facilitating cellular internalization *via* receptor-mediated endocytosis; and (iii) activatable targeting from endogenous GSH-induced active drug release in cancer cells.

Accurate tracking of *in vivo* tumor-specific behavior with probes is a perfect strategy for targeted sensing and controlled release of prodrugs.^1^–^3^ However, a significant limitation of such theranostic nanoprobe design is that multiple components for such a complicated scheme are often required, inevitably leading to structural heterogeneity, insufficient reproducibility and subsequently huge barriers for clinical translation.^4^ Current reporting strategies mainly focus on physical entrapment or chemical modification of drugs with multicomponent nanocarriers, including polymers,^5^–^15^ liposomes^16^–^23^ and inorganic materials.^24^–^32^ With the help of these nanovehicles, probes with prolonged blood circulation duration and enhanced permeability and retention (EPR effect) show more effective and specific cancer treatment than free drugs.^33^–^42^ However, the inevitable leakage and non-uniform loading efficiency based on the physical encapsulation system are insuperable barriers (Fig. 1a). In contrast, polymer–drug conjugates offer other notable benefits to reduce premature leakage.^43^–^49^ But the critical issue with polymer–drug conjugates is polydispersity in both the degree of polymerization and extent of loading attachment with chemical means (Fig. 1a). This inherent structural heterogeneity could cause significant batch-to-batch variability, which is an impassable obstacle for clinical translation. Even worse, almost all current theranostics suffer from limitations that imaging and therapy are independently performed, rather than in an integrated protocol.^50^ Thus, structural heterogeneity and the discrete steps of imaging and therapy make an unpredictable gap between how drugs behave *in vitro* and *in vivo*, that is, great difficulties in real-time tracking of drug release and evaluating therapeutic efficacy. To address these hurdles, the design of monodisperse nanomaterials with a single, reproducible entity that possess both *in vivo* diagnostic and therapeutic competencies is highly in demand.

Herein, we describe the rational design strategy of molecularly precise amphiphilic nanotheranostics by using functional building blocks. The main idea of this approach is to construct a structurally homogenous prodrug in a single, reproducible entity with synergistic targeting ability, which integrates the advantageous features of small molecular theranostics and polymer–drug conjugates (Fig. 1a). It makes full use of the two terminal conjunctions of the bis-condensed dicyanomethylene-4*H*-pyran (DCM) derivative as the activatable near-infrared (NIR) fluorophore: the hydrophobic disulfide-bridged anticancer drug camptothecin (CPT) and the hydrophilic PEG oligomer-bridged biotin segment serving as an active targeting unit. We focus on optimizing the hydrophilic fragment length to construct stable, well-defined nanostructured assemblies. Specifically, these amphiphilic structures of biotin-PEG_*n*_-DCM-S-CPT or BP_*n*_-DCM-S-CPT (Fig. 1) are composed of PEG_*n*_-biotin units (*n* = 0, 5 and 20) as the controllable hydrophilic fragments and the covalently linked hydrophobic DCM-S-CPT moiety as the fluorescent reporter. Notably, only BP_20_-DCM-S-CPT could spontaneously form uniform, stable, and reproducible core–shell micellar nanostructures (Fig. 1b). More importantly, the shell surface-grafted biotin directly exposed to receptors on cancer cells can markedly facilitate cellular internalization. As demonstrated, these molecularly precise amphiphilic nanoprodrugs possess several striking characteristics: (i) well-defined monodisperse nanostructures with excellent reproducibility, high stability and fixed loading efficiency; (ii) real-time tracking of active drug release; (iii) synergistic passive (preferable micelle-based EPR effect), active (biotin receptor-mediated endocytosis) and activatable (endogenous GSH-induced active drug release) targeting ability with extremely high inhibition rates of tumour growth (IRT). As far as we know, BP_20_-DCM-S-CPT is the first molecularly precise self-assembled nanotheranostics which can be implemented for *in situ* and *in vivo* tracking of antitumor chemotherapy in living animal models.

## Results and discussion

### Rational design of molecularly precise theranostic nanoprobes within a single-molecular framework by tuning PEG segments

**Scheme 1:**
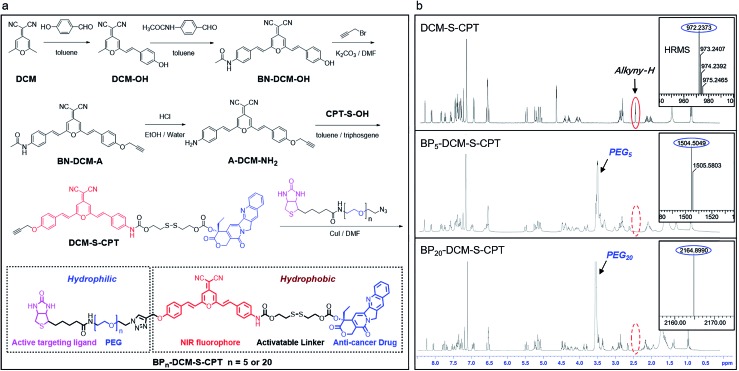
Optimizing the hydrophilic PEG fragment length for molecularly precise prodrugs. (a) Synthetic route to DCM-S-CPT, BP_5_-DCM-S-CPT and BP_20_-DCM-S-CPT. (b) ^1^H NMR and high resolution mass spectra (HRMS) of DCM-S-CPT, BP_5_-DCM-S-CPT and BP_20_-DCM-S-CPT. Note: the excellent reproducibility, structural homogeneity and fixed loading efficiency of BP_*n*_-DCM-S-CPT completely avoid the batch-to-batch variability of the physical encapsulation system and polymer–drug conjugates.

In our system, all intrinsic building blocks are considered as both functionally active and structurally guiding units. The functional PEG_*n*_-biotin unit as a hydrophilic shell is not only used to stabilize the micelles and prolong the blood circulation time but also acts as an active targeting ligand resulting in receptor-mediated endocytosis^51^ and enhanced uptake into tumor cells.^52^,^53^ Bis-condensed dicyanomethylene-4*H*-pyran (DCM) is employed as the fluorescent reporter owing to its attractive features such as controllable emission wavelength in the NIR region, large Stokes shift, high photostability (Fig. S1a†), and particularly making full use of the two terminal reactive conjunctions.^54^,^55^ The synthetic route to BP_*n*_-DCM-S-CPT is depicted in Scheme 1. A-DCM-NH_2_ was initially reacted with the key intermediate CPT-S-OH in the presence of triphosgene at room temperature. Finally, BP_*n*_-DCM-S-CPT (*n* = 5 and 20) prodrugs were obtained by the reaction of DCM-S-CPT with the azido-PEG_*n*_-biotin moiety *via* a typical ‘click’ reaction in a yield of 30%. Specifically, in the ^1^H NMR spectra (Scheme 1b), the propargyl proton (*δ* = 2.57 ppm) of DCM-S-CPT disappeared during the formation of BP_*n*_-DCM-S-CPT, while new protons (*δ* = 3.56–3.68 ppm) corresponding to PEG chains (–CH_2_–) were observed. Moreover, the two peaks at *m*/*z* 1504.5049 (corresponding to [BP_5_-DCM-S-CPT + H]^+^) and 2164.8990 (corresponding to [BP_20_-DCM-S-CPT + H]^+^) clearly further identify these molecularly precise structures from their individual high resolution mass spectra (HRMS, Scheme 1b). All the detailed procedures and characterizations are shown in the ESI.†

As is well known, conventional carrier-based nanodrugs suffer from variable drug contents and release capacities from batch-to-batch. In contrast, molecularly precise self-delivery prodrugs have significant advantages of fixed and higher drug loadings. In our case, the fixed drug content of BP_*n*_-DCM-S-CPT (*n* = 0, 5 and 20) was directly calculated from its molecular structure, corresponding to the respective drug loadings of 36%, 23% and 16% (Table S1†). These well-defined structural prodrugs can feature constant drug release capacities. The excellent reproducibility, structural homogeneity and fixed loading efficiency of BP_*n*_-DCM-S-CPT completely avoid the inherent structural drawback of polymer–drug conjugates (Fig. 1a).

### Critical effect of PEG segment lengths on forming highly stable amphiphilic micelle-based nanotheranostics

**Fig. 2:**
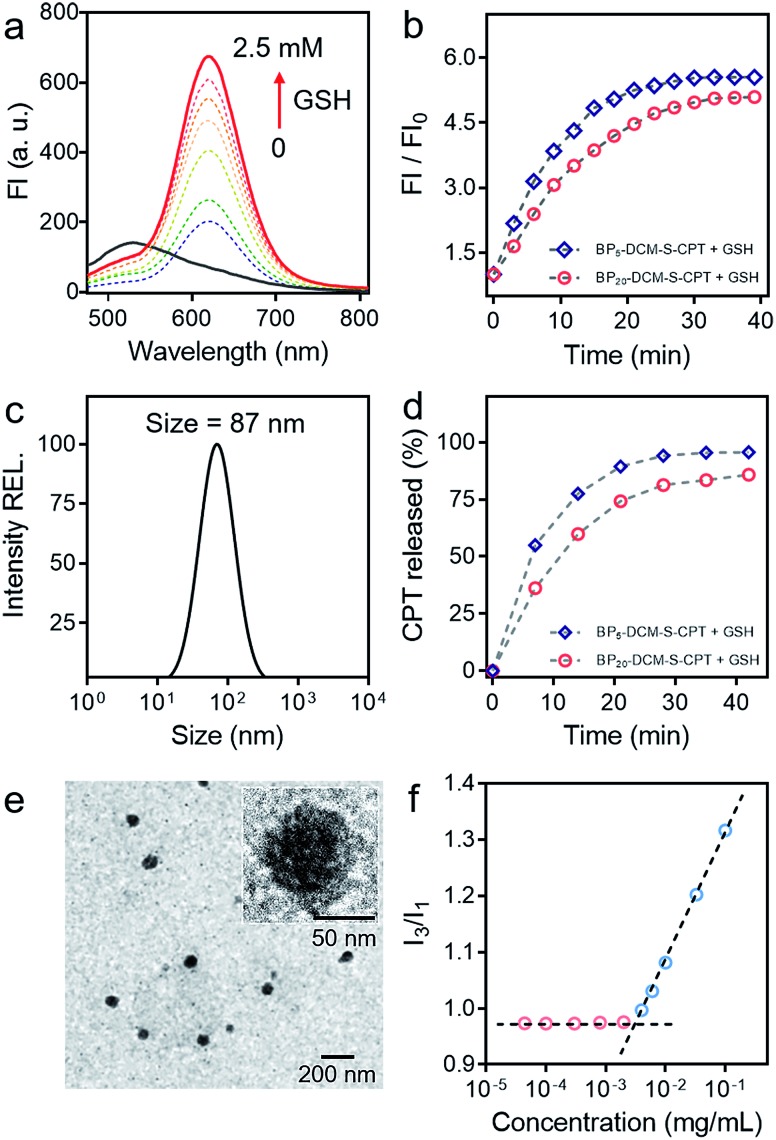
Stable amphiphilic core–shell micellar self-assembly and GSH stimulus-response. (a) Emission spectral changes of DCM-S-CPT (10 μM) in the presence of GSH (each point was recorded after exposure to GSH for 1 h at 37 °C), *λ*_ex_ = 480 nm. (b) Time dependence of fluorescence intensity at 650 nm for BP_5_-DCM-S-CPT (molecular dissolution state) and BP_20_-DCM-S-CPT (self-assembled micellar state) with GSH (2.5 mM) in PBS. (c) Size distribution of BP_20_-DCM-S-CPT (10 μM). (d) CPT released from BP_5_-DCM-S-CPT and BP_20_-DCM-S-CPT as a function of time with GSH (2.5 mM) HPLC chromatograms. (e) TEM images of BP_20_-DCM-S-CPT. Inset: the amplified image. (f) Critical micelle concentration of BP_20_-DCM-S-CPT (3.3 μg mL^–1^). Note: GSH-induced active drug release is always in synchronism with NIR fluorescence signals either in the self-assembled micellar state or molecular dissolution state.

As a unifying rule, when the ratio of hydrophilic moiety to total mass *f*_hl_ in amphiphilic molecules is over 0.45, they can be expected to form stable micelles. The inherent amphiphilicity and suitable *f*_hl_ (0.56) of BP_20_-DCM-S-CPT provide itself an opportunity to self-assemble into nanoparticles in aqueous solution.^56^ To determine the size, morphology and stability of the self-assembled nanoparticles, a dimethyl sulfoxide (DMSO) solution of BP_20_-DCM-S-CPT was added dropwise into water, followed by dialysis against water to remove DMSO. A stable solution with a final BP_20_-DCM-S-CPT conjugate concentration of 1.0 mg mL^–1^ was obtained. Notably, we observed that the assemblies of BP_20_-DCM-S-CPT had an average size of 87 nm measured by dynamic laser scattering (DLS) with a PDI (polydispersion index) of 0.25 (Fig. 2c). The size and morphology of the self-assembled nanostructures were further confirmed by transmission electron microscopy (TEM). As shown in Fig. 2e, TEM images reveal that the BP_20_-DCM-S-CPT micelles have uniform spherical shapes with an average size of approximately 70 nm. This size corresponds to that measured by DLS but only slightly smaller due to the drying stage during the TEM sample preparation.

Notably, the DLS measurements at different time intervals during 32 days demonstrated that these assemblies exhibited extremely high stability for long storage (Fig. S1b†). Moreover, the surface charge of the BP_20_-DCM-S-CPT solution was also investigated. The results show that the value of zeta potential is –3.9 mV, a lower negative surface charge, thus causing less repulsion between NPs and the cell membrane in aqueous solution. Most importantly, these well-defined BP_20_-DCM-S-CPT self-assemblies maintain their good initial stability over 96 h in fresh human serum at 37 °C (Fig. S2 and S3†). To gain further insight into its self-assembly behavior in aqueous solution, the critical micelle concentration (CAC) of BP_20_-DCM-S-CPT was measured by using pyrene as a fluorescent probe, and the results are shown in Fig. 2f. Notably, the CAC value of BP_20_-DCM-S-CPT is calculated to be only about 3.3 μg mL^–1^ (1.52 μM), which is much lower than those of reported amphiphilic self-assembled systems.^57^

In contrast, when decreasing the PEG oligomer chain from 20 to 0 or 5, it became difficult for the resulting DCM-S-CPT and BP_5_-DCM-S-CPT to form stable self-assembled micelles, which was further confirmed by TEM (Fig. S4a†). These observations clearly indicated that the regulation of the suitable hydrophilic fragment length of PEG_*n*_-biotin units in BP_*n*_-DCM-S-CPT is very important to form stable, well-defined nanostructured assemblies. Definitely, it is of critical importance to balance hydrophobicity and hydrophilicity for the formation of well-defined nanostructures. In this case, we successfully build the strategy of molecularly precise amphiphilic self-assembly of prodrugs, wherein all the building blocks including the hydrophobic DCM-S-CPT moiety and hydrophilic PEG_*n*_-biotin units are vital structurally guiding elements rather than just functional units.

### Activatable targeting: GSH-induced active drug release in synchronism with NIR fluorescence signals

Owing to its D–π–A structure, DCM-S-CPT exhibits a typical broad ICT absorption band at 455 nm with fairly weak emission at 529 nm in a mixed DMSO/PBS buffer solution (40/60, v/v, pH = 7.4, 10 mM, Fig. 2a). On the other hand, A-DCM-NH_2_ has strong broad fluorescence under the same conditions. The distinct difference between A-DCM-NH_2_ and DCM-S-CPT in the spectroscopic properties can be attributed to the masking of the electron-withdrawing amide bond in DCM-S-CPT, along with disruption of the electron donating ability of the nitrogen atom.^55^,^58^ Notably, the emission band of A-DCM-NH_2_ is broad so that A-DCM-NH_2_ still has a very strong fluorescence signal over 650 nm, which guarantees A-DCM-NH_2_ as a promising fluorophore for *in vivo* imaging.

First of all, to test our system containing an activatable disulfide linker, the spectral properties of the prodrug DCM-S-CPT upon reaction with GSH were investigated in a mixed DMSO/PBS solution (40/60, v/v, pH = 7.4, 10 mM). As expected, the prodrug DCM-S-CPT produced fluorescence spectral changes as shown in Fig. 2a. Upon treatment with 2.5 mM GSH, significant NIR fluorescence was activated, which increased by about 10-fold upon excitation at 480 nm. Subsequently, the anticipated release of CPT as an active cancer drug was further proven by ESI-MS analyses. In the presence of GSH, the peaks of DCM-S-CPT at *m*/*z* 453.1 (corresponding to [CPT-SH + H]^+^), 522.2 (corresponding to [A-DCM-SH + H]^+^) and 418.2 (corresponding to [A-DCM-NH_2_ + H]^+^) were simultaneously observed in the HRMS (Fig. S5†). Clearly, it was indicated that the active CPT can be released from the prodrug DCM-S-CPT upon exposure to GSH, with concomitant generation of the NIR fluorescent reporter A-DCM-NH_2_ by a two-step reaction (cleavage of the disulfide bond and then intramolecular cyclization, Scheme S2†).^59^ Correspondingly, similar results of BP_*n*_-DCM-S-CPT (*n* = 5 and 20) were also observed in the emission spectra (Fig. S6b†). All these pieces of evidence clearly indicated that these prodrugs could be specifically activated by abundant GSH in aqueous solution.

Subsequently, the effect of self-assembled micellar nanostructure formation on GSH-induced active drug release was systematically studied. In our strategy, the consistent supply of bioactive CPT from the self-assembled nanostructures in the presence of GSH is anticipated to be directly visualized using fluorescent reporter signals. As expected, in its molecular dissolution state, the time-course experiments of BP_*n*_-DCM-S-CPT (*n* = 5 and 20) in the presence of GSH were found to correlate well with the continuous increase in fluorescence intensity at 650 nm (arising from the activated DCM moiety in Fig. S6†). Notably, in PBS solution (pH = 7.4), the well-defined micellar assemblies of BP_20_-DCM-S-CPT exhibited slower responses towards GSH than BP_5_-DCM-S-CPT (Fig. 2b). Furthermore, TEM of BP_20_-DCM-S-CPT in aqueous solution demonstrated the formation of micelles in the absence of GSH (Fig. 2e) and complete micelle dissociation in the presence of GSH (Fig. S4b†), suggesting the active drug release induced by GSH. This observation suggested that the PEG_20_-biotin unit of BP_20_-DCM-S-CPT as the hydrophilic shell could reduce permeation of GSH into the activatable disulfide-bridged hydrophobic core, which might diminish premature release side-effects.

Either in the self-assembled micellar state or a molecular dissolution state, neither CPT release by HPLC nor the fluorescence enhancement of BP_20_-DCM-S-CPT was observed in the absence of GSH. As shown in Fig. S7,† upon addition of GSH, both the CPT release and fluorescence enhancement of BP_20_-DCM-S-CPT in the molecular dissolution state reached a plateau within 30 min. In contrast, it took more than 35 min to reach the same plateau in the self-assembled state of BP_20_-DCM-S-CPT (Fig. 2d). Most importantly, we found that upon GSH-triggering, the consistent CPT release of self-assembled BP_20_-DCM-S-CPT (Fig. S8†) is always synchronized with its continuous NIR fluorescence enhancement in PBS solution. This could be attributed to molecularly precise BP_20_-DCM-S-CPT with a single, reproducible entity. All these pieces of evidence confirmed that this molecularly precise BP_20_-DCM-S-CPT has the key characteristics of the CPT release in synchronism with NIR fluorescence enhancement. Thus, the turn-on NIR fluorescence signal from the self-assembly of BP_20_-DCM-S-CPT with a single, reproducible entity could be used to precisely monitor the active CPT release in the presence of GSH, performing the activatable targeting function.

Having confirmed the GSH-driven NIR response of BP_20_-DCM-S-CPT in buffer solution, we then assessed whether these nanoassemblies can be used in real biological systems. The *in vitro* release behaviour of BP_20_-DCM-S-CPT with other biologically relevant amino acids, enzymes and serum markers was investigated (Fig. S9†). Upon exposure to 1,4-dithiothreitol (DTT), cysteine (Cys), and homocysteine (Hcy), a similar spectroscopic response of DCM-S-CPT to GSH could be observed due to its thiol-containing structure. On the other hand, no appreciable fluorescence enhancement could be induced by treatment with other non-thiol amino acids, enzymes and serum markers, confirming the specific cleavage of the disulphide bond elicited by thiol-containing species. Actually, the potential interference of DTT, Cys, and Hcy can be neglected due to their relatively low concentration in contrast to that with a high physiological concentration of GSH in the cytoplasm. Meanwhile, the BP_20_-DCM-S-CPT nanostructures exhibited enough high stability in an aqueous solution or fresh human serum with uniform size, which is ideal for tumor targeting by means of the EPR effect. All these results indicated that BP_20_-DCM-S-CPT can sustain in the inactive form under normal physiological conditions but is capable of consistently releasing active CPT in synchronism with turn-on NIR fluorescence signals under the GSH-overexpressed physiological conditions.

### Active targeting: shell surface-grafted biotin directly exposed to receptors on cancer cells for facilitating cellular internalization and visualizing drug release in living cells

**Fig. 3:**
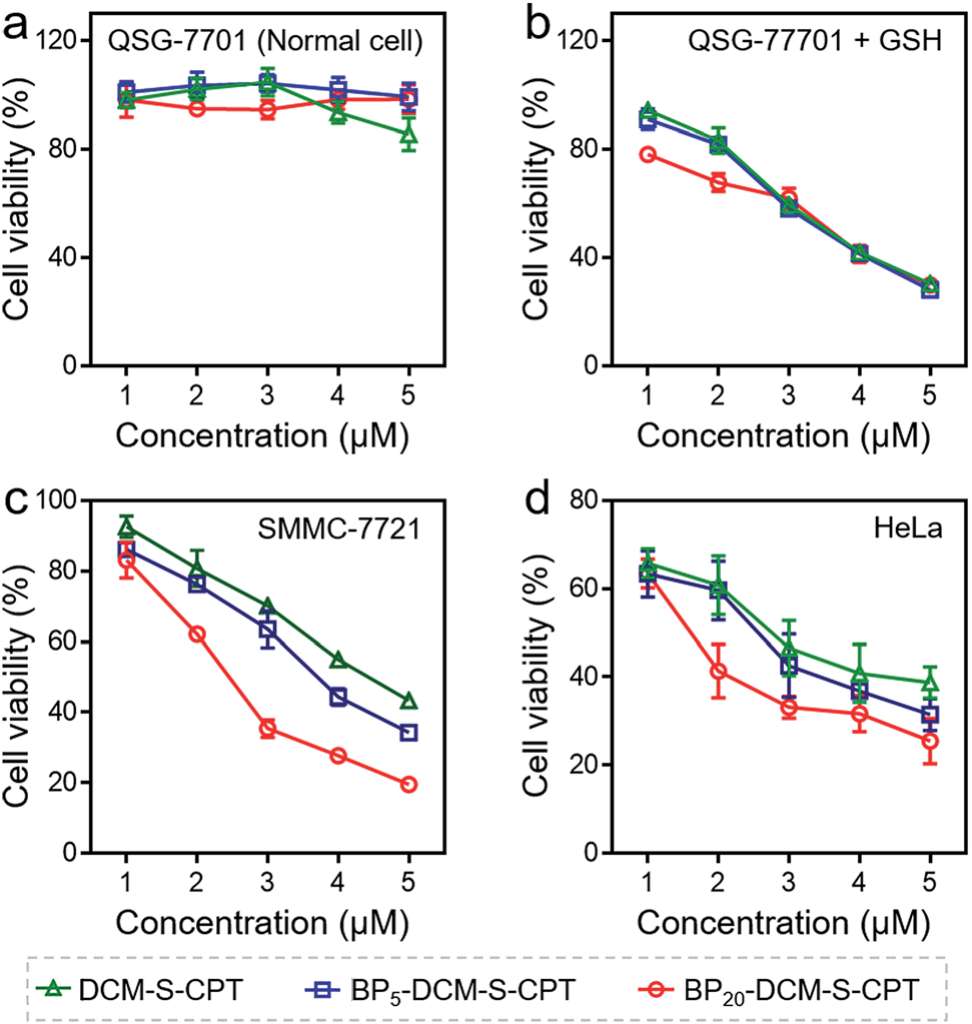
Nanoassembling effect on *in vitro* cytotoxicity with the MTT assay. Human hepatocyte QSG-7701 cells (a), QSG-7701 cells with extra 2.5 mM GSH (b), human hepatoma SMMC-7721 cancer cells (c) and HeLa cancer cells (d) were incubated with various concentrations (0–5 μM) of DCM-S-CPT, BP_5_-DCM-S-CPT and BP_20_-DCM-S-CPT for 24 h. Data are shown as mean ± s.d., with *n* = 3. Note: shell surface-grafted biotin of BP_20_-DCM-S-CPT directly exposed to receptors on cancer cells markedly facilitates cancer cell seeking and killing.

The *in vitro* toxicity of our designed molecularly precise prodrugs BP_*n*_-DCM-S-CPT (*n* = 0, 5 and 20) was assessed by using the standard MTT assay. A number of cell lines including normal cells (QSG-7701) and cancer cells (SMMC-7721 and HeLa) were chosen to incubate with BP_*n*_-DCM-S-CPT. As depicted in Fig. 3a and S10,† all BP_*n*_-DCM-S-CPT prodrugs exhibit no cytotoxic effects on normal cells at the studied concentrations (0–20 μM). In contrast, the remarkably higher cytotoxicity of BP_*n*_-DCM-S-CPT in the presence of GSH was observed with cancer tumor cells, indicating that the released bioactive CPT by GSH is dominant and mainly responsible for the observed cellular toxicity (Fig. 3b). Meanwhile, all these prodrugs BP_*n*_-DCM-S-CPT exhibited significantly higher cytotoxicity for cancer cells (SMMC-7721 and HeLa) because of abundant intracellular GSH in cancer cells (Fig. 3c and d).^60^–^62^ Furthermore, upon addition of extra GSH, a further enhancement of cytotoxicity was found in cancer cells (Fig. S11†). In conjunction with the above results, we can conclude that the observed toxicity mainly results from the GSH-triggered released active CPT inducing cell apoptosis.

**Fig. 4:**
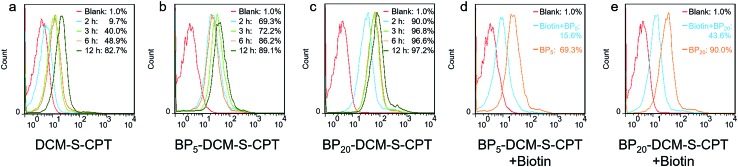
Targeted cellular internalization *via* biotin receptor mediated endocytosis. Flow cytometry analysis of cellular uptake of DCM-S-CPT (a), BP_5_-DCM-S-CPT (b) and BP_20_-DCM-S-CPT (c) at different time intervals from 2 to 12 h in HeLa cells. Flow cytometry analysis of cellular uptake of BP_5_-DCM-S-CPT (d) and BP_20_-DCM-S-CPT (e) in the presence of competing free biotin. Note: compared with the competing test, the shell surface-grafted biotin markedly facilitates cellular internalization of cancer cells.

In fact, the recognition and binding efficiency of biotin and biotin receptors is the key factor that significantly influences cancer cell seeking and cellular internalization.^63^ As can be imagined, the shell surface-grafted biotin of BP_20_-DCM-S-CPT directly exposed to the receptors on cancer cells can markedly facilitate cellular internalization *via* biotin receptor-mediated endocytosis. Thus, the effect of self-assembled nanostructure formation on the toxicity of prodrugs was also investigated. Notably, the general trends of BP_*n*_-DCM-S-CPT were clearly observed in cancer cells (SMMC-7721 and HeLa cells) that the sharply enhanced cytotoxicity was found with varying concentrations from 1 to 5 μM (Fig. 3c and d). Specifically, the BP_20_-DCM-S-CPT assemblies exhibited the highest cytotoxicity in cancer cells, while BP_5_-DCM-S-CPT showed higher cytotoxicity in contrast to DCM-S-CPT. This could be mainly attributed to the more exposed biotin-grafted surface targeting units with the core–shell micellar nature of BP_20_-DCM-S-CPT nanostructures (Fig. 1b). Taken together, these results validated that a higher cytotoxicity of BP_20_-DCM-S-CPT assemblies resulted from the more effective uptake by cancer cells. Cancer cell specific uptake is one of the important factors affecting therapeutic efficacy in tumor therapy. To understand whether the internalization of our self-assembled prodrugs might be improved, the properties of BP_*n*_-DCM-S-CPT were further evaluated in HeLa cancer cells by flow cytometry analysis. In fact, it is well-known that biotin receptors are overexpressed on many cancer cell surfaces, for example HeLa cells.^64^ As shown in Fig. 4a, it took 12 h for the cellular uptake ratios of DCM-S-CPT to increase from 9.7% to 82.7%. In contrast, within only 3 h, the uptake ratios of BP_5_-DCM-S-CPT and BP_20_-DCM-S-CPT increased to 72.2% and 96.8%, respectively, meaning that the biotin units of these two prodrugs effectively increased the uptake efficacy of cancer cells (Fig. 4b and c).

To further verify shell surface-grafted biotin facilitating cellular internalization, HeLa cells were pretreated with free biotin so that the overexpressed biotin receptor on the HeLa cell surface was mostly bound. Subsequently, we observed the slower uptake ratios of BP_5_-DCM-S-CPT and BP_20_-DCM-S-CPT (15.6% and 43.6%) within 3 h, respectively (Fig. 4d and e). All these findings are thus fully consistent with our design concept of BP_*n*_-DCM-S-CPT, that is, incorporation with biotin unit can be uptake into biotin receptor-positive cancer cells with high efficiency. In particular, the shell surface-grafted biotin on the BP_20_-DCM-S-CPT self-assemblies markedly facilitates cellular internalization of cancer cells.

**Fig. 5:**
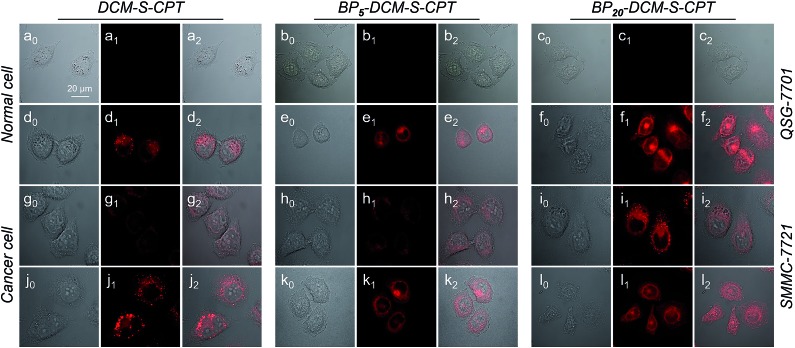
GSH-driven drug release in synchronism with NIR fluorescence signals in living cells. Confocal laser scanning microscopy images (*λ*_ex_ = 488 nm and *λ*_em_ = 650–700 nm) of QSG-7701 cells (a–c), QSG-7701 cells with extra 2.5 mM GSH (d–f), SMMC-7721 cells (g–i) and SMCC-7721 cells with extra 2.5 mM GSH (j–l) incubated with DCM-S-CPT, BP_5_-DCM-S-CPT and BP_20_-DCM-S-CPT. Channel 0: bright field; Channel 1: fluorescence signal of activated prodrugs; Channel 2: overlapped field. Note: with the help of DCM as the activatable NIR fluorophore, BP_20_-DCM-S-CPT micellar self-assemblies can successfully achieve the effective cellular uptake and quickly converted into emissive BP_20_-DCM-NH_2_ and active CPT by endogenous GSH.

Based on the tumor-specific intracellular uptake, the GSH-induced disulfide linkage cleavage in concomitance with turn-on NIR fluorescence in BP_*n*_-DCM-S-CPT offers an opportunity for directly visualizing the *in vitro* drug release by confocal laser scanning microscopy. After 3 h incubation with cells at 37 °C, only for cancer cells, the turn-on NIR fluorescence from BP_*n*_-DCM-S-CPT was found in the cytoplasm (Fig. 5g–i), suggesting that the endogenous GSH triggers the active CPT release. Notably, of the three prodrugs, BP_20_-DCM-S-CPT exhibited the strongest turn-on fluorescence signal in cancer cells. It is firmly evident that the BP_20_-DCM-S-CPT self-assemblies showed the most effective cellular uptake and quickly converted into emissive BP_20_-DCM-NH_2_ and active CPT by endogenous GSH (Fig. 5i). Consistent with flow cytometry and the *in vitro* cytotoxicity by the MTT assay, all these fluorescence measurements provided solid evidence that the BP_20_-DCM-S-CPT micellar assemblies are intrinsically suited for tumor targeted delivery and controlled release of CPT to tumor cells. We can attribute that the shell surface-grafted biotin guaranteed the highly efficient cellular internalization of cancer cells (Fig. 1b). Thus, our strategy of molecularly precise self-assembly of theranostics within a single, reproducible entity provides great opportunities to gain insight into the mechanisms of their dynamic assemblies and targeted therapies.

### Synergistic targeting: *in situ* behavior of self-assembled amphiphilic prodrugs in living animals

**Fig. 6:**
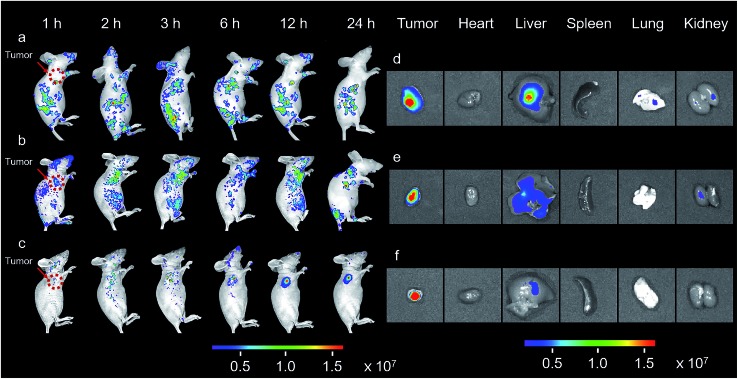
*In vivo* and *in situ* imaging of BP_*n*_-DCM-S-CPT. *In vivo* NIR-fluorescence imaging of HeLa xenograft tumor-bearing mice at various times (1, 2, 3, 6, 12 and 24 h) after intravenous injection of DCM-S-CPT (a), BP_5_-DCM-S-CPT (b) and BP_20_-DCM-S-CPT (c) administered at a CPT-equivalent dose. The red arrow indicates the tumor site. *Ex vivo* NIR-fluorescence imaging of excised organs (tumor, heart, liver, spleen, lung and kidney) at 24 h after the intravenous injection of DCM-S-CPT (d), BP_5_-DCM-S-CPT (e) and BP_20_-DCM-S-CPT (f). The color bars correspond to the detected fluorescence intensity. Note: real-time tracking of the *in vivo* behavior of prodrugs confirmed that BP_20_-DCM-S-CPT self-assemblies have synergistic passive, active and activatable targeting efficiency for tumor seeking and specific uptake.

The promising results in living cells such as specifically seeking cancer cells and facilitating cellular internalization inspired us to further explore the feasibility of BP_*n*_-DCM-S-CPT as an *in vivo* NIR fluorescence-tracking and synergistic targeting drug delivery system. The *in vivo* drug delivery performance and biodistribution of BP_20_-DCM-S-CPT, BP_5_-DCM-S-CPT and DCM-S-CPT were investigated with HeLa tumor-bearing mice after intravenous injection at different time intervals, respectively. The nude mice were inoculated with HeLa cells on their right flanks by injecting 10^6^ cells subcutaneously. As shown in Fig. 6a, the biodistribution profiles show that only a small amount of fluorescence of DCM-S-CPT was located at the tumor site, while there was strong fluorescence in the liver. In contrast, BP_5_-DCM-S-CPT with active targeting biotin exhibited an enhanced amount of tumor accumulation (Fig. 6b). In particular, due to the specific self-assembled micelle with shell-grafted biotin, BP_20_-DCM-S-CPT possessed both passive targeting from the EPR effect and active targeting, exhibiting a significantly enhanced tumor accumulation after 24 h injection (Fig. 6c).

The *ex vivo* fluorescence images of excised tumors further confirmed the highest accumulation of BP_20_-DCM-S-CPT assemblies, with much weaker fluorescence in the liver and no fluorescence in the heart, spleen, lung and kidney (Fig. 6f). In contrast, DCM-S-CPT and BP_5_-DCM-S-CPT exhibited strong fluorescence signals in the liver region, suggestive of a continuous CPT release triggered by the liver metabolism function towards small molecular prodrugs (Fig. 6d and e). In the case of BP_20_-DCM-S-CPT, the rational hydrophilic PEG length is very critical to form micelles, which can greatly facilitate passive EPR targeting and active targeting *via* the more exposed biotin-grafted surface. Here all the synergistic targeting (passive targeting from the EPR effect, active targeting from shell grafted biotin, and activatable endogenous GSH-driven targeting) makes BP_20_-DCM-S-CPT achieve high carrying efficiency, good targeting properties, and sustained and tumor-specific release in living mice.

### *In vivo* anticancer activity with high tumor growth inhibition rates

**Fig. 7:**
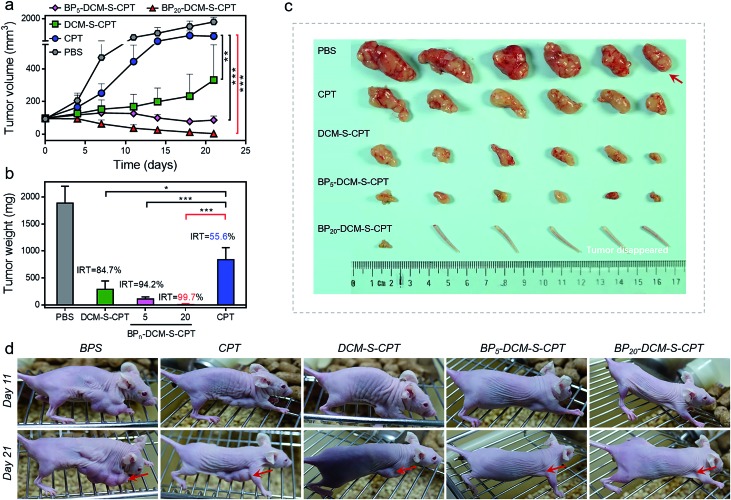
*In vivo* anticancer activity. (a) Changes of the tumor volume of HeLa xenograft tumor-bearing mice after intravenous injection of PBS, CPT, DCM-S-CPT, BP_5_-DCM-S-CPT and BP_20_-DCM-S-CPT administered at a CPT-equivalent dose of 10 mg kg^–1^ every 3 days; each tumor was measured at the time of the injection and its relative tumor volume was calculated (*n* = 6, data expressed as average ± standard error). On day 21, the mice were terminated, and the tumors were dissected and weighed (statistical significance: *P* values, **p < 0.01, ***p < 0.001, compared with the CPT group are calculated by the *t* test). (b) Mean weight of tumors separated from mice after different treatments and the inhibition rates of tumor growth (IRT) after treatment with CPT, DCM-S-CPT, BP_5_-DCM-S-CPT and BP_20_-DCM-S-CPT in HeLa tumor-bearing nude mice. The IRT is calculated using the following equation: IRT (%) = 100 × (mean tumor weight of control group – mean tumor weight of experimental group)/mean tumor weight of control group (*n* = 6, data expressed as average ± standard error; statistical significance: *P* values, *p < 0.05, and ***p < 0.001, compared with the CPT group are calculated by the *t* test). (c) Representative tumors separated from animals after intravenous injection of PBS, CPT, DCM-S-CPT, BP_5_-DCM-S-CPT and BP_20_-DCM-S-CPT. Part of the tail indicates that the solid tumor completely disappeared after treatment. (d) The mice are treated with different formulations, and the tumor size is real-time monitored during the 21-day evaluation period. Note: synergistic passive, active and activatable targeting ability of BP_20_-DCM-S-CPT self-assemblies provides them with impressive performance of high tumor growth inhibition rates and *in vivo* anticancer activity.

To evaluate whether precise targeting and improved biodistribution result in the enhancement of therapeutic efficacy, the *in vivo* anticancer activities were compared using the HeLa xenograft tumor model in nude mice. The mice were randomly divided into six groups (*n* = 6) in order to minimize weight and tumor-size differences. Mice bearing the tumors were intravenously injected with CPT, DCM-S-CPT, BP_5_-DCM-S-CPT and BP_20_-DCM-S-CPT at a CPT-equivalent dose of 10 mg kg^–1^ and phosphate buffer solution (PBS) as a control *via* the tail vein. The tumor volume and body weight of HeLa tumor-bearing mice were monitored every 3 days for 21 days (Fig. 7a). CPT, DCM-S-CPT, BP_5_-DCM-S-CPT and BP_20_-DCM-S-CPT could inhibit HeLa tumor growth compared with the blank control group. As shown in Fig. 7b, compared with that of the PBS group, the inhibition rates of tumor growth (IRT) of CPT, DCM-S-CPT, BP_5_-DCM-S-CPT and BP_20_-DCM-S-CPT on HeLa tumors were 58.7%, 85.8%, 94.5%, and 99.7%, respectively. All BP_*n*_-DCM-S-CPT show low toxicity with no significant weight loss during the treatment throughout the experiments, indicating that our designed prodrugs DCM-S-CPT, BP_5_-DCM-S-CPT and BP_20_-DCM-S-CPT groups did not cause severe systematic side effects, whereas CPT could lead to toxicity in mice (Fig. S13†).

Obviously, BP_20_-DCM-S-CPT exhibited the highest antitumor efficacy, revealing the priority of passive targeting (EPR), active targeting and activatable targeting to achieve the highest tumor accumulation and carrying efficiency. In addition, BP_5_-DCM-S-CPT group treatment led to significant inhibition of tumor growth compared to DCM-S-CPT (Fig. 7c), illustrating a better drug delivery efficacy *via* active targeting biotin. It's worth noting that BP_20_-DCM-S-CPT nearly eradicates the tumor (Fig. 7c and d), suggesting that BP_20_-DCM-S-CPT almost cures the mice with cancer bearing tumors. These data verify that the specific nanoassemblies of BP_20_-DCM-S-CPT exerted excellent therapeutic activity in synchronism with *in vivo* turn-on NIR fluorescence biodistribution *via* synergistic targeting in living animals.

## Conclusions

In summary, we described the rational design strategy of molecularly precise self-assembled nanotheranostics for *in situ* and *in vivo* tracking of antitumor chemotherapy, in which PEG_*n*_-biotin units are utilized as the tunable hydrophilic fragments and the hydrophobic DCM-S-CPT moiety as an activatable NIR fluorescent reporter. It is found that the hydrophilic PEG length is very critical to form the stable micellar nanoassemblies. Upon changing different PEG oligomers from 0 to 5 and 20, only BP_20_-DCM-S-CPT can simultaneously self-assemble into uniform micelles (*ca.* 80 nm) with a low CAC value, which displays high stability in fresh human serum. As demonstrated, the molecularly precise self-assembled nanotheranostics BP_20_-DCM-S-CPT can not only overcome the inevitable drug leakage and non-uniform drug payload based on the physically encapsulated drug delivery system, but also avoid the polydispersity in both the degree of polymerization and extent of drug loading in the polymer–drug conjugate system.

The well-defined monodisperse nanoassemblies of BP_20_-DCM-S-CPT possess the unique feature of real-time tracking of active CPT release in synchronism with turn-on NIR fluorescence signals. BP_20_-DCM-S-CPT displays excellent tumor site-specific delivery in HeLa tumor-bearing nude mice *via* synergistic targeting, including passive targeting from the EPR effect, active biotin targeting (shell surface-grafted biotin directly exposed to receptors on cancer cells markedly facilitates cellular internalization), and endogenous GSH-induced specific cleavage. As a result, BP_20_-DCM-S-CPT displays high tumor growth inhibition rates and *in vivo* anticancer activity and nearly eradicates the tumor (IRT = 99.7%). In particular, the *in situ* synergistic targeting behavior of self-assembled amphiphilic prodrugs in living animals can overcome the limitations in the current theranostics, that is, imaging and therapy are independently performed, rather than in an integrated protocol. These *in vivo* and *in situ* behaviors with molecularly precise self-assembled theranostics make significant insight into understanding amphiphilic self-delivery nanotheranostics, presenting new opportunities to drug-loading nanostructures.

## Conflicts of interest

There are no conflicts to declare.

## Supplementary Material

Supplementary information
