# Supplementary material for: Molecularly precise self-assembly of theranostic nanoprobes within a single-molecular framework for in vivo tracking of tumor-specific chemotherapy
Source: Chem Sci. 2018 Apr 24;9(22):4959–69. doi: 10.1039/c8sc01069b (PMC5989654; doi:10.1039/c8sc01069b)
Supplement: Supplementary file 1 [file SC-009-C8SC01069B-s001.pdf]

Electronic Supplementary Material (ESI) for Chemical Science.
This journal is © The Royal Society of Chemistry 2018

Electronic Supplementary Information (ESI)

Molecularly precise self-assembling theranostic nanoprobe within single-molecular framework for *in vivo* tracking tumor-specific chemotherapy

Chenxu Yan,[a] Zhiqian Guo,*[a] Yanyan Shen,[b] Yi Chen,[b] He Tian[a] and Wei-Hong Zhu*[a]

[a]Key Laboratory for Advanced Materials and Institute of Fine Chemicals, Shanghai Key Laboratory of Functional Materials Chemistry, East China University of Science and Technology, Shanghai 200237, China.

[b]Division of Anti-Tumor Pharmacology, State Key Laboratory of Drug Research, Shanghai Institute of Materia Medica, Chinese Academy of Sciences, Shanghai 201203, China

E-mail: whzhu@ecust.edu.cn; guozq@ecust.edu.cn

Contents

**1.**	**Experimental Section**	**S3-S10**
**2.**	**Stability of BP20-DCM-S-CPT micelle**	**S11-S12**
**3.**	**TEM Image of BP_5-DCM-S-CPT and BP_{20}-DCM-S-CPT & GSH**	**S12**
**4.**	**Proposed Mechanism of BPn-DCM-S-CPT Reaction with GSH**	**S13**
**5.**	**ESI-MS Spectra Characterization of DCM-S-CPT Reaction with GSH**	**S13**
**6.**	**Effect of Self-Assembled Nanostructure Formation on GSH-Induced Drug Release**	**S14**
**7.**	**HPLC Chromatogram of BP20-DCM-S-CPT**	**S15**
**8.**	**Selectivity of BP20-DCM-S-CPT**	**S15**
**9.**	**Cytotoxicity of DCM-S-CPT, BP5-DCM-S-CPT and BP20-DCM-S-CPT**	**S16**
**10**	**Confocal Laser Scanning Microscopy Images of HeLa Cells**	**S16**
**11.**	**Antitumor Activity of BPn-DCM-SCPT**	**S17-S18**
**12.**	**Characterization of Intermediate Compounds and BPn-DCM-S-CPT**	**S19-S28**

1. Experimental Section

Materials and General Methods

Unless special stated, all solvents and chemicals were purchased from commercial suppliers in analytical grade and used without further purification. Biotin-PEG$_5$-N$_3$ and Biotin-PEG$_{20}$-N$_3$ was supplied by Biomatrik Inc. (Jiaxing, China). The ^{1}H and ^{13}C NMR spectra were recorded on a Bruker AM 400 spectrometer, using TMS as an internal standard. High resolution mass spectrometry data were obtained with a Waters LCT Premier XE spectrometer. Absorption spectra were collected on a Varian Cary 500 spectrophotometer, and fluorescence spectra measurements were performed on a Varian Cary Eclipse fluorescence spectrophotometer. Transmission electron microscopy (TEM) images were obtained on a JEOL 100CX transmission electron microscope operating at an accelerating bias voltage of 100 kV. Particle size was measured by dynamic light scattering (DLS) with a NICOMP 380 ZLS. HPLC analysis was performed on an Agilent 1100 series. Confocal fluorescence images were taken on confocal laser scanning microscope (CLSM, Nikon A1R/A1). Flow cytometric analysis was carried out with a FACSCalibur flow cytometer (BD Biosciences, Franklin Lakes, NJ, USA). *In vivo* fluorescence images in *vivo* fluorescence images were measured with a PerkinElmer IVIS Lumina Kinetic Series III imaging system.

Synthesis of DCM-S-CPT, BP$_5$-DCM-S-CPT and BP$_{20}$-DCM-S-CPT

The intermediate compound CPT-S-OH[1] was synthesized by the established procedures.

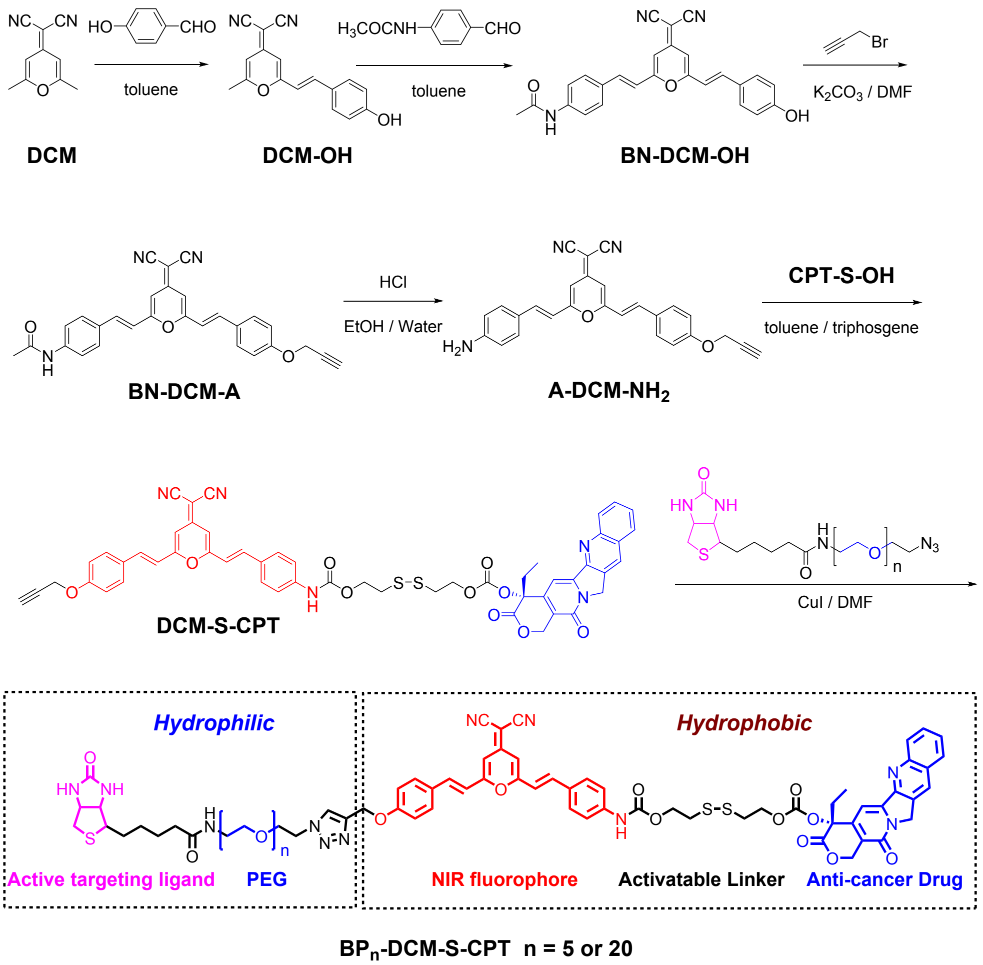

Scheme S1. Synthetic route of DCM-S-CPT, BP$_5$-DCM-S-CPT and BP$_{20}$-DCM-S-CPT

Synthesis of DCM-OH

DCM (1.04 g, 6.0 mmol) and 4-hydroxybenzaldehyde (0.36 g, 2.96 mmol) were dissolved in toluene (30 mL) with acetic acid (0.5 mL) and piperidine (1.0 mL). Then the mixture was refluxed for 10 h under an argon atmosphere. The solvent was removed under reduced pressure, and then the crude product was purified by silica gel chromatography using dichloromethane/ethyl acetate/PE (v/v/v, 1:1:1) as the eluent to afford DCM-OH as a yellow solid (184 mg): Yield 23%. ^{1}H NMR (400 MHz, DMSO-d_6, ppm) δ: 2.45 (s, 3H, -CH_3), 6.68 (s, 1H, ph-H), 6.83 (d, 3H, ph-H), 7.14 (d, 1H, J = 16.0 Hz, alkene-H), 7.47 (d, 1H, J = 16.0 Hz, alkene-H), 7.56 (d, 2H, J = 8.0 Hz, ph-H), 10.08 (s, 1H, -OH). ^{13}C NMR (100 MHz, DMSO-d_6, ppm): δ 163.94, 160.46, 159.71, 156.71, 137.82, 129.97, 125.92, 115.92, 115.57, 115.39, 105.90, 105.67, 54.98, 19.36. Mass spectrometry (ESI-MS, m/z): $[M - H]^-$ calcd for $C_{17}H_{11}N_2O_2$, 275.0821; found, 275.0818

Synthesis of BN-DCM-OH

DCM-OH (100 mg, 0.36 mmol) and N-(4-formylphenyl) acetamide (118 mg, 0.72 mmol) were dissolved in dry toluene (30 mL) with acetic acid (0.5 mL) and piperidine (1.0 mL). Then the mixture was refluxed for 10 h under an argon atmosphere. The solvent was removed under reduced pressure, and then the crude product was purified by silica gel chromatography using dichloromethane/methyl alcohol (v/v, 50:1) as the eluent to afford BN-DCM-OH as a red solid (110 mg): Yield 72%. ^{1}H NMR (400 MHz, DMSO-d_6, ppm) δ: 2.08 (s, 3H, -CH_3), 6.81 (d, 2H, J = 10.0 Hz, ph-H), 6.85 (d, 2H, J = 8.6 Hz, ph-H), 7.17 (d, 1H, J = 16.0 Hz, alkene-H), 7.28 (d, 1H, J = 16.0 Hz, alkene-H), 7.68(d, 5H, J = 10.0 Hz, ph-H), 7.75(d, 3H, J = 8.8 Hz, ph-H), 10.09 (s, 1H, -OH), 10.18 (s, 1H, -NH). ^{13}C NMR (100 MHz, DMSO-d_6, ppm): δ 167.46, 158.85, 158.39, 157.85, 154.93, 139.95, 137.10, 136.09, 129.07, 128.56, 127.82, 124.90, 117.72, 116.91, 116.29, 114.77, 114.63, 114.30, 105.38, 104.78, 54.38, 22.98. Mass spectrometry (ESI-MS, m/z): $[M - H]^-$ calcd for $C_{26}H_{18}N_3O_3$, 420.1348; found, 420.1354.

Synthesis of A-DCM-NH_2

BN-DCM-OH (200 mg, 0.48 mmol), 3-bromoprop-1-yne (115 mg, 0.96 mmol) and K_2CO_3 (130 mg, 0.96 mmol) were dissolved in dry DMF (15 mL). Then the reaction mixture was stirred overnight at room temperature under an argon atmosphere. The solution was added with DCM (50 mL) and washed with water (50 mL × 3), dried over Na_2SO_4, filtered and evaporated to BN-DCM-A. Mass spectrometry (ESI-MS, m/z): $[M - H]^-$ calcd for $C_{29}H_{20}N_3O_3$, 458.1505; found, 458.1510. BN-DCM-A were dissolved in conc. HCl and ethanol (2:1, 30 mL). Then the mixture was refluxed for 5 h under an argon atmosphere. The aqueous solution was extracted with ethyl acetate and then the organic layers were dried over Na_2SO_4, filtered, and concentrated to obtain the crude product. Then the crude product was purified by silica gel chromatography using dichloromethane/methyl alcohol (v/v, 100:1) as the eluent to afford A-DCM-NH_2 as a red solid (70 mg): Yield 35%. ^{1}H NMR (400 MHz, DMSO-d_6, ppm) δ: 3.64 (s, 1H, alkynyl-H), 4.89 (s, 2H, -NH_2), 5.93 (s, 2H, -CH_2-), 6.62 (d, 2H, J = 8.4 Hz, ph-H), 6.71 (s, 1H, pyran-H), 6.79 (s, 1H, pyran-H), 7.01 (d, 1H, J = 16.0 Hz, alkenyl-H), 7.10 (d, 2H, J = 8.4 Hz, ph-H), 7.28 (d, 1H, J = 16.0 Hz, alkenyl-H), 7.54 (d, 2H, J = 8.4 Hz, ph-H), 7.66 (d, 1H, J = 16.0 Hz, alkenyl-H), 7.71 (d, 1H, J = 16.0 Hz, alkenyl-H), 7.80 (d, 2H, J = 8.4 Hz, ph-H). ^{13}C NMR (100 MHz, DMSO-d_6, ppm): δ 167.45, 158.66, 157.83, 154.91, 139.95, 136.95, 136.07, 129.05, 128.54, 127.81, 124.99, 117.70, 116.26, 114.72, 114.61, 114.39, 105.36, 104.79, 80.52, 75.42, 74.85, 54.44. Mass spectrometry (ESI-MS, m/z): $[M + H]^+$ calcd for $C_{27}H_{20}N_3O_2$, 418.1556; found, 418.1555.

Synthesis of DCM-S-CPT

To a mixture of A-DCM-NH_2 (100 mg, 0.24 mmol), triphosgene (287 mg, 0.95 mmol) and dry toluene (30 mL) was added DIEA (500 mg, 3.9 mmol) dropwise under an argon atmosphere at room temperature. The resulting solution was refluxed under argon protection for 3 h. After removal of unreacted phosgene gas by flushing argon gas, a solution of CPT-S-OH (253 mg, 0.48 mmol) in $CHCl_3$ (10 mL) was added to the mixture and the reaction mixture was stirred overnight at room temperature. After removing the solvent under reduced pressure, the crude product was purified by silica gel chromatography using ethyl acetate/PE (v/v, 1:1) as the eluent to afford DCM-S-CPT as a yellow solid (88 mg): Yield 38%. ^{1}H NMR (400 MHz, $CDCl_3$, ppm) δ: 1.03 (t, *J* = 7.5 Hz, 3H, -CH_3), 2.10-2.17 (m, 1H, -**CH_2**CH_3), 2.22-2.31 (m, 1H, -**CH_2**CH_3), 2.57 (t, *J* = 2.3 Hz, 1H, alkynyl-H), 2.92-3.06 (m, 4H, -CH_2-S-S-CH_2-), 4.09-4.15 (m, 1H, -CH_2), 4.18-4.24 (m, 1H, -CH_2), 4.39-4.45 (m, 1H, -CH_2), 4.51-4.57 (m, 1H, -CH_2), 4.77 (d, *J* = 2.3 Hz, 2H, O-CH_2-), 5.17-5.31 (m, 2H,-NCH_2-), 5.35 (d, *J* = 17.1 Hz, 1H), 5.55 (d, *J* = 17.1 Hz, 1H), 6.65 (d, *J* =13.2 Hz, 2H, ph-H), 6.69 (d, *J* =13.2 Hz, 2H, ph-H), 7.35 (s, 1H, ph-H),7.40 (d, *J* =8.6 Hz, 2H, ph-H), 7.47 (d, *J* =8.4 Hz, 2H, ph-H), 7.50-7.53 (m, 3H, ph-H), 7.57 (d, *J* =8.7 Hz, 2H, ph-H), 7.69 (t, *J* = 7.3 Hz, 1H, ph-H), 7.86 (t, *J* = 7.1 Hz, 1H, ph-H), 7.96 (d, *J* = 8.1 Hz, 1H, ph-H), 8.23 (d, *J* = 8.6 Hz, 1H, ph-H), 8.41 (s, 1H, ph-H). ^{13}C NMR (100 MHz, DMSO-*d*$_6$, ppm): *δ* 167.45, 165.89, 158.60, 158.33, 157.84, 155.31, 154.91, 151.65, 151.04, 146.72, 145.09, 143.58, 139.96, 136.95, 136.09, 130.41, 129.25, 129.04, 128.59, 127.82, 127.34, 126.84, 126.57, 125.03, 118.02, 117.72, 116.90, 116.25, 114.72, 114.60, 114.42, 105.36, 104.79, 93.21, 80.25, 76.72, 76.41, 65.29, 65.16, 58.13, 54.46, 53.74, 49.14, 39.96, 29.17, 6.41. Mass spectrometry (ESI-MS, m/z): $[M + H]^+$ calcd for $C_{53}H_{42}N_5O_{10}S_2$, 972.2373; found, 972.2373.

Synthesis of BP_5-DCM-S-CPT

To a mixture of DCM-S-CPT (30 mg, 0.031 mmol), Biotin-PEG$_5$-N_3 (25 mg, 0.046 mmol) and dry DMF (2 mL) was added CuI (12 mg, 0.060 mmol) dropwise under an argon atmosphere at room temperature. Then the reaction mixture was stirred overnight at room temperature. The solution was added with DCM (20 mL) and washed with water (20 mL × 5), dried over Na_2SO_4, filtered and evaporated to dryness. Then the crude product was purified by silica gel chromatography using dichloromethane/methyl alcohol (v/v, 10:1) as the eluent to afford BP_5-DCM-S-CPT as a yellow solid (12 mg): Yield 26%. ^{1}H NMR (400 MHz, $CDCl_3$, ppm) δ: 8.43 (s, 1H, ph-H), 8.25 (d, *J* = 8.5 Hz, 1H, ph-H), 7.97 (t, *J* = 8.1 Hz, 1H, ph-H), 7.87 (t, *J* = 7.2 Hz, 1H, ph-H), 7.73 (s, 1H, ph-H), 7.70 (t, *J* = 7.5 Hz, 1H, ph-H), 7.85 (d, *J* = 7.0 Hz, 2H, ph-H), 7.53 (d, *J* = 8.3 Hz, 3H, ph-H), 7.48 (d, *J* = 6.7 Hz, 2H, ph-H), 7.44 (d, *J* = 8.3 Hz, 2H, ph-H), 7.36 (s, 1H, ph-H), 7.13 (d, *J* = 8.7 Hz, 1H, ph-H), 7.10 (d, *J* = 8.4 Hz, 1H, ph-H), 6.76-6.66 (m, 4H, ph-H), 5.63 (d, *J* = 17.1 Hz, 1H, alkene-H), 5.37 (d, *J* = 17.1 Hz, 1H, alkene-H), 5.33-5.22 (m, 4H, ph-H), 4.61-4.60 (m, 2H, -CH_2), 4.56-4.51 (m, 2H, -CH_2), 4.46-4.40 (m, 1H, -CH), 4.35-4.31 (m, 1H, -CH), 4.25-4.16 (m, 2H, -S-CH_2), 4.00-3.90 (m, 2H, -CH_2), 3.68-3.56 (m, 16H, -O-CH_2), 3.48-3.40 (m, 4H, -CH_2), 3.04-2.91 (m, 6H, -CH_2), 2.74 (d, *J* = 12.7 Hz, 1H, -CH), 2.31-2.14 (m, 4H, -CH_2), 1.70-1.67 (m, 4H, -CH_2), 1.48-1.45 (m, 2H, -CH_2), 1.04 (t, 3H, *J* = 7.3 Hz, -CH_3). ^{13}C NMR (100 MHz, DMSO-*d*$_6$, ppm): *δ* 170.97, 167.46, 165.89, 161.57, 158.60, 158.30, 157.80, 155.30, 154.87, 151.66, 151.01, 146.72, 145.08, 143.59, 139.96, 136.92, 136.06, 130.40, 129.24, 129.03, 128.55, 127.80, 127.33, 126.83, 126.55, 125.02, 118.01, 117.71, 116.90, 116.22, 114.71, 114.59, 114.39, 105.34, 104.78, 93.21, 76.72, 68.64, 68.57, 68.55, 68.42, 68.10, 68.02, 65.16, 59.89, 58.13, 58.05, 54.48, 54.28, 48.84, 39.95, 37.30, 35.01, 33.94, 29.17, 27.04, 26.88, 24.11, 22.98, 6.41. Mass spectrometry (ESI-MS, m/z): $[M + H]^+$ calcd for $C_{75}H_{82}N_{11}O_{17}S_3$, 1504.5052; found, 1504.5049.

Synthesis of BP_{20}-DCM-S-CPT

To a mixture of DCM-S-CPT (30 mg, 0.031 mmol), Biotin-PEG_{20}-N_3 (54 mg, 0.046 mmol) and dry DMF (2 mL) was added CuI (12 mg, 0.060 mmol) dropwise under an argon atmosphere at room temperature. Then the reaction mixture was stirred overnight at room temperature. The solution was added with DCM (20 mL) and washed with water (20 mL × 5), dried over Na_2SO_4, filtered and evaporated to dryness. Then the crude product was purified by silica gel chromatography using dichloromethane/methyl alcohol (v/v, 10:1) as the eluent to afford BP_{20}-DCM-S-CPT as a yellow solid (20 mg): Yield 30%. ^{1}H NMR (400 MHz, $CDCl_3$, ppm) δ: 8.42 (s, 1H, ph-H), 8.23 (d, J = 8.6 Hz, 1H, ph-H), 7.96 (d, J = 8.0 Hz, 1H, ph-H), 7.91 (s, 1H, ph-H), 7.86 (t, J = 6.9 Hz, 1H, ph-H), 7.69 (t, J = 7.1 Hz, 1H, ph-H), 7.63 (d, J = 6.5 Hz, 1H, ph-H), 7.58-7.55 (m, 2H, ph-H), 7.53-7.51 (m, 3H, ph-H), 7.47 (d, J = 5.9 Hz, 2H, ph-H), 7.42 (d, J = 8.5 Hz, 2H, ph-H), 7.35 (s, 1H, ph-H), 7.12 (d, J = 8.8 Hz, 1H, ph-H), 7.09 (d, J = 8.8 Hz, 1H, ph-H), 6.70 (d, J = 13.9 Hz, 1H, alkene-H), 6.67 (s, 2H, ph-H), 6.66 (d, J = 11.6 Hz, 1H, alkene-H), 5.61 (d, J = 17.1 Hz, 1H, alkene-H), 5.36 (d, J = 17.0 Hz, 1H, alkene-H), 5.28-5.19 (m, 4H, ph-H), 4.60-4.55 (m, 2H, -CH_2), 4.53-4.49 (m, 2H, -CH_2), 4.45-4.39 (m, 1H, -CH), 4.34-4.31 (m, 1H, -CH), 4.26-4.12 (m, 2H, -S-CH_2), 3.97 (t, J = 5.8 Hz, 1H, -CH), 3.89 (t, J = 5.0 Hz, 1H, -CH), 3.65-3.62 (m, 76 H, -O-CH_2), 3.56 (t, J = 5.1 Hz, 4H, -CH_2), 3.45 (t, J = 5.2 Hz, 4H, -CH_2), 3.18-3.13 (m, 1 H, -CH), 3.02-2.90 (m, 5 H, -CH_2), 2.73 (d, J = 12.7 Hz, 1H, -CH), 2.34-2.12 (m, 4H, -CH_2), 1.72-1.60 (m, 4H, -CH_2), 1.49-1.40 (m, 2H, -CH_2), 1.03 (t, J = 7.4 Hz, 3H, -CH_3). ^{13}C NMR (100 MHz, DMSO-d_6, ppm): δ 168.58, 167.01, 162.67, 159.73, 159.45, 158.96, 156.43, 156.02, 152.77, 152.15, 147.84, 146.21, 144.71, 141.08, 138.07, 137.21, 131.54, 130.38, 130.16, 129.70, 129.66, 128.94, 128.46, 127.96, 127.70, 126.15, 119.14, 118.83, 117.36, 115.84, 115.71, 115.52, 106.47, 105.91, 94.33, 77.84, 69.75, 69.54, 69.23, 69.15, 66.27, 61.00, 59.25, 59.16, 55.58, 55.40, 49.96, 41.07, 38.41, 36.13, 35.05, 30.28, 28.16, 28.01, 25.23, 24.09, 7.53. Mass spectrometry (ESI-MS, m/z): $[M + H]^+$ calcd for $C_{105}H_{142}N_{11}O_{32}S_3$, 2164.8985; found, 2164.8990.

Preparation of BP_{20}-DCM-S-CPT Nanoparticles

In a typical procedure for the preparation of BP_{20}-DCM-S-CPT nanoparticles: 10 mg BP_{20}-DCM-S-CPT was dissolved in 1.0 mL of DMSO and stirred at room temperature (25 ^{o}C) for 10 min. Then the mixture was slowly added into 9.0 mL of deionized water and stirred slightly for another 10 min. Subsequently, the solution was dialyzed against deionized water for 24 h (molecular weight cutoff = 1,000 g mol^{-1}) and the deionized water was exchanged for 4 times.

Drug Loading Calculations

The drug loading of the drug amphiphiles is given as the CPT weight as a percentage of the total molecular weight:

$$Drug\ loading\ (\%) = \frac{347.1}{M_{DA}} \times 100\ \%$$

where M_{DA} is the exact mass of the prodrugs.

$$DCM - CPT\ Drug\ loading\ (\%) = \frac{347.1}{972.2} \times 100\ \% = 35.7\ \%$$

$$BP_5 - DCM - CPT\ Drug\ loading\ (\%) = \frac{347.1}{1504.5} \times 100\ \% = 23.1\ \%$$

$$BP_{20} - DCM - CPT\ Drug\ loading\ (\%) = \frac{347.1}{2164.9} \times 100\ \% = 16.0\ \%$$

Table S1. Molecular Weights, Drug Loading Content, Water Solubility, Concerted Targeting and Nanostructure of Amphiphilic Prodrugs

Amphiphilic Prodrugs	M_{HRMS} (Da)[a]	DLC/%[b]	Water Solubility	Activatable Targeting	Active Targeting	Passive Targeting	Nanostructure
DCM-S-CPT	972.2373	35.7	-	√	-	-	-
BP_5-DCM-S-CPT	1504.5049	23.1	√	√	√	-	*Cannot form stable self-assemblies*
BP_{20}-DCM-S-CPT	2164.8990	16.0	√	√	√	√	*Highly Stable self-assemblies (ca. 80 nm)*

[a]Molecular weights M_{HRMS} were evaluated by high resolution mass spectrometry. [b]Drug loading content (DLC) was calculated as the ratio of exact molecular weight of CPT to the exact molecular weight of amphiphilic prodrugs.

Transmission Electron Microscopy (TEM) Protocol

All 10 µM sample solutions were prepared from a stock solution of 1 mM in water. Samples were aged overnight prior to sample preparation. Samples were prepared by depositing 10 µL of the appropriate solution onto a carbon-coated copper grid (Electron Microscopy Services, Hatfield, PA), wicking away the excess solution with a small piece of filter paper. The sample grid was then allowed to dry at room temperature (25 °C) prior to imaging. TEM imaging was performed on a JEOL 100CX transmission electron microscope operating at an accelerating bias voltage of 100 kV.

CMC Measurements

Critical micelle concentration (CMC) value is the amphiphilic prodrugs concentration at which micelles would form in solution. CMC of BP_{20}-DCM-S-CPT in the 0.2 M sodium phosphate buffer at pH 7.4. First, the stock solution (5 mg/ml) was diluted to different concentrations with the same buffer. In each solution, 5 µL pyrene in THF solution (2×10^{-4} M) was added to 2 ml BP_{20}-DCM-S-CPT solution to produce the final pyrene concentration at 5×10^{-7} M. The fluorescence spectra were recorded with the excitation wavelength of 339 nm and the excitation. The I_1 and I_3 values were measured as the maximum emission intensity at ca. 372 and 382 nm, respectively.

I_3/I_1 ratio was plotted as a function of polymer concentration. I_3/I_1 ratio reflects the polarity of the pyrene environment where partition of pyrene in the hydrophobic micelle core leads to increased I_3/I_1 values.

Cell Experiment

Cell Lines

The human hepatocyte QSG-7701 cell line, human hepatoma SMMC-7721 cell line and human epithelioid cervical carcinoma cell line HeLa were purchased from the Institute of Cell Biology (Shanghai, China). Cells were all propagated in T-75 flasks cultured at 37 ºC under a humidified 5% CO_2 atmosphere in RPMI-1640 medium or DMEM medium (GIBCO/Invitrogen, Camarillo, CA, USA), which were supplemented with 10 % fetal bovine serum (FBS, Biological Industry, Kibbutz Beit Haemek, Israel) and 1 % penicillin-streptomycin (10,000 U mL^{-1} penicillin and 10 mg/ml streptomycin, Solarbio life science, Beijing, China).

In Vitro Cytotoxicity Assay

The cell cytotoxicity of DCM-S-CPT, BP_5-DCM-S-CPT and BP_{20}-DCM-S-CPT to QSG-7701 cells, SMMC-7721 cells and HeLa cells were measured by 3-(4, 5-dimethylthiazol-2-yl)-2, 5-diphenyltetrazolium bromide (MTT) assay. The cytotoxicity was evaluated by Cell Counting Kit-8 (Dojindo, Tokyo, Japan) according to the factory's instruction. Cells were plated in 96-well plates in 0.1 mL volume of DMEM or RPMI-1640 medium with 10 % FBS, at a density of 1×10^4 cells/well and added with desired concentrations of DCM-S-CPT, BP_5-DCM-S-CPT and BP_{20}-DCM-S-CPT. After incubation for 24 h, absorbance was measured at 450 nm with a Tecan GENios Pro multifunction reader (Tecan Group Ltd., Maennedorf, Switzerland). Each concentration was measured in triplicate and used in three independent experiments. The relative cell viability was calculated by the equation: cell viability (%) = $(OD_{treated}/OD_{control}) \times 100\%$

In Vitro Cytotoxicity Assay Pre-Treated with GSH

Cells were plated in 96-well plates in 0.1 mL volume of DMEM or RPMI-1640 medium with 10 % FBS, at a density of 1×10^4 cells/well and added with desired concentrations of GSH. After incubation for 3 h, PBS (pH 7.4) was used to washed cells for three times to clean the extracellular GSH. Then the cells were exposed to desired concentrations of DCM-S-CPT, BP_5-DCM-S-CPT and BP_{20}-DCM-S-CPT for 24 h with complete medium. After incubation for 24 h, absorbance was measured at 450 nm with a Tecan GENios Pro multifunction reader (Tecan Group Ltd., Maennedorf, Switzerland). Each concentration was measured in triplicate and used in three independent experiments. The relative cell viability was calculated by the equation: cell viability (%) = $(OD_{treated}/OD_{control}) \times 100\%$

Measurement of Intracellular GSH Levels

To measure the intracellular glutathione (GSH) concentration, QSG-7701 cells, SMMC-7721 cells pre-treated with and without GSH were cultured and prepared. The intracellular GSH from cell extract can react with DTNB (5,5-dithio-bis-[2-nitrobenzenic acid]) to form the coloured GSH-DTNB conjugate, which can be determined by the change in absorbance at 412 nm.

In Vitro Cellular Imaging

The QSG-7701 cells and SMMC-7721 cells at 1×10^5 cells/well were seeded onto glass-bottom petri dishes with complete medium (1.5 mL) for 12 h. Then the cells pre-incubated with and without GSH were exposed to desired concentrations of DCM-S-CPT, BP_5-DCM-

S-CPT and BP_{20}-DCM-S-CPT for 3 h. PBS (pH 7.4) was used to washed cells for three times to clean the background. 4 % paraformaldehyde was added at room temperature for 20 min. The fixed cells were rinsed with PBS (pH 7.4) twice. The images were then photographed by using a Nikon A1R confocal laser scanning microscope with 488 nm as the excitation wavelength and 650-700 nm as the emission wavelength.

GSH-Dependent Intracellular Fluorescent Release by Flow Cytometry Assay

The HeLa cells at 2×10^5 cells/well were seeded onto six-well plates with 2 mL complete medium and cultured overnight. The cells were then treated with the designed concentrations of Biotin, DCM-S-CPT, BP_5-DCM-S-CPT and BP_{20}-DCM-S-CPT at 37℃ for 2 h, 3h, 6 h or 12 h. After washed with PBS twice, the cells were harvested using 0.05 % (w/v) trypsin/0.02 % (w/v) EDTA. The collected cells were resuspended in PBS (500 μL) after washed with cold PBS twice. Each sample was measured with flow cytometry (FACSCalibur) within 1 h (λex = 488 nm, 680/20 nm bandpass filter).

Animals

All animal studies were approved by the Animal Care and Use Committee of Shanghai Institute of Materia Medica in accordance with the guidelines for the care and use of laboratory animals. The 3-4-week-old female BALB/cA nude mice were producted from Shanghai Institute of Materia Medica, Chinese Academy of Sciences, and maintained under standard conditions. The animals were housed in sterile cages within laminar airflow hoods in a specific pathogen-free room with a 12-h light/12-h dark schedule and fed autoclaved chow and water *ad* libitum. Number of qualitative qualification: No.311613700000141. Production Permit No.: SCXK (Shanghai) 2013-0017. SYXK No. of Shanghai Institute of Materia Medica: SYXK (Shanghai) 2013-0049.

Real-Time *in Vivo* Imaging in Tumor-Bearing Mice

The nude mice were inoculated with HeLa cell on their right flanks by injecting 10^6 cells subcutaneously. When the tumors grew up to 10 mm in diameter, DCM-S-CPT, BP_5-DCM-S-CPT and BP_{20}-DCM-S-CPT (administered at a CPT-equivalent dose of 0.1 mg/kg) in PBS were intravenously injected via tail vein into the HeLa cell tumor-bearing nude mice. The real-time *in vivo* imaging *in vivo* imaging was recorded at different time internals after DCM-S-CPT, BP_5-DCM-S-CPT and BP_{20}-DCM-S-CPT injection using PerkinElmer IVIS Lumina Kinetic Series III imaging system. The concentration of the injected solution is 3 μM (PBS, pH = 7.4). In the in vivo imaging, λ_{ex} = 470 nm, λ_{em} = 650 nm. After injection, the mice were sacrificed at 24 h. The grafted tumor tissues and major organs, including kidney, lung, spleen, liver, and heart, were excised and washed with 0.9% saline. The optical images of the organs and tissues were taken using a PE *in vivo* Professional Imaging System as described above.

In Vivo Antitumor Studies

To develop the human tumor xenografts, HeLa cells were implanted into the right flanks of mice (5.0×10^6 cells). When the tumors reached a mean volume of around 90-100 mm^3, mice were randomly assigned to five treatment groups (n = 6/group): (a) PBS, (b) camptothecin (CPT) at 10 mg/kg, (c) DCM-S-CPT, (d) BP_5-DCM-S-CPT and (e) BP_{20}-DCM-S-CPT at equivalent dose of 10 mg/kg CPT. The mices were intravenously injected with PBS, CPT, DCM-S-CPT, BP_5-DCM-S-CPT and BP_{20}-DCM-S-CPT via the tail vein every 3 days, repeated for 3 weeks. Each animal was earmarked and followed individually throughout the experiment. Tumor volume

(mm^3) was calculated by V (mm^3) = 1/2 × A (mm) × B $(mm)^2$, where A and B were respectively the longest and widest diameters of tumor. The curve of tumor growth was drawn based on tumor volume (mm^3) and corresponding time (days). Animals were sacrificed after the 3 weeks treatment according to institutional guidelines. Tumors were resected, weighed. To evaluate the therapeutic efficacy of the treatment, inhibition rates (IRT) of tumor growth were calculated using the following equation: IRT = 100 % × (mean tumor weight of control group - mean tumor weight of experimental group)/mean tumor weight of control group.

2. Stability of BP_{20}-DCM-S-CPT

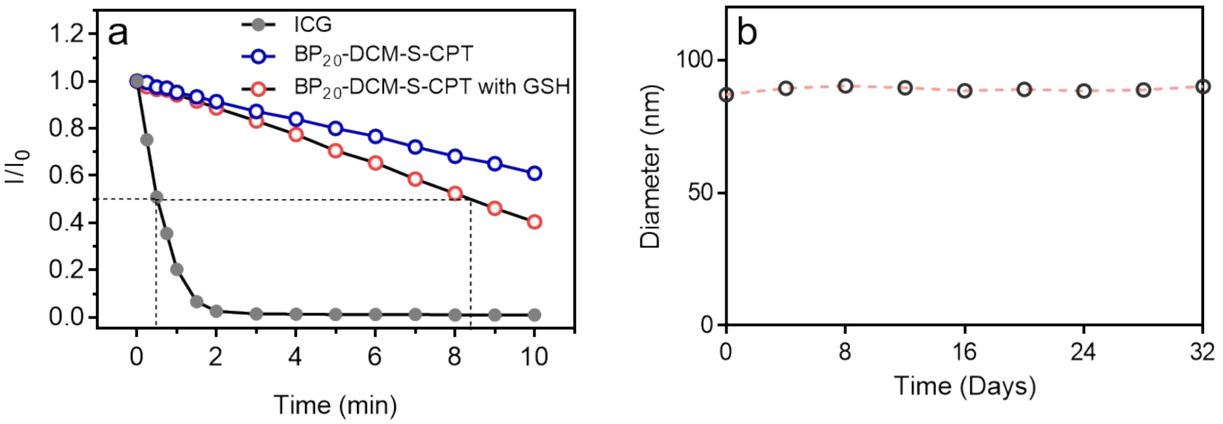

Fig. S1 (a) Time-dependent fluorescence intensity of ICG (10 μM, monitored at 812 nm), BP_{20}-DCM-S-CPT (10 μM, treated with 2.5 mM GSH for 1 h at 37 °C, monitored at 650 nm), and BP_{20}-DCM-S-CPT (10 μM, monitored at 650 nm) under illumination. (b) BP_{20}-DCM-S-CPT self-assemblies remain stable in PBS (pH 7.4) over 32 days at 37 °C.

The photostability of NIR fluorophores in drug delivery systems is a crucial element for practical application in bioimaging *in vivo*. The time-dependent fluorescence measurements were conducted for the photostability of GSH treated BP_{20}-DCM-S-CPT upon continuous illumination (Hamamatsu, LC8 Lightningcure, 300 W), and compared with cyanine dye ICG (the FDA-approved NIR contrast agent). After exposure for about 100 s, the fluorescence of ICG decreased sharply to approximate 5% of the initial value (monitored at 812 nm), indicative of he almost complete decomposition of the scaffold of ICG, while for BP_{20}-DCM-S-CPT in the presence of GSH, more than 80% of the original fluorescence at 650 nm survived. Obviously, BP_{20}-DCM-S-CPT on treatment with GSH as well as production of BP_{20}-DCM-NH_2 exhibited much better photostability than ICG. As calculated from the time-course fluorescence measurements, the fluorescence half-life of BP_{20}-DCM-S-CPT (~505 s) is 19-fold longer than that of ICG (~27 s). The high photostability of BP_{20}-DCM-S-CPT indicates that it can be used as an excellent contrast agent for *in vivo* bioimaging.

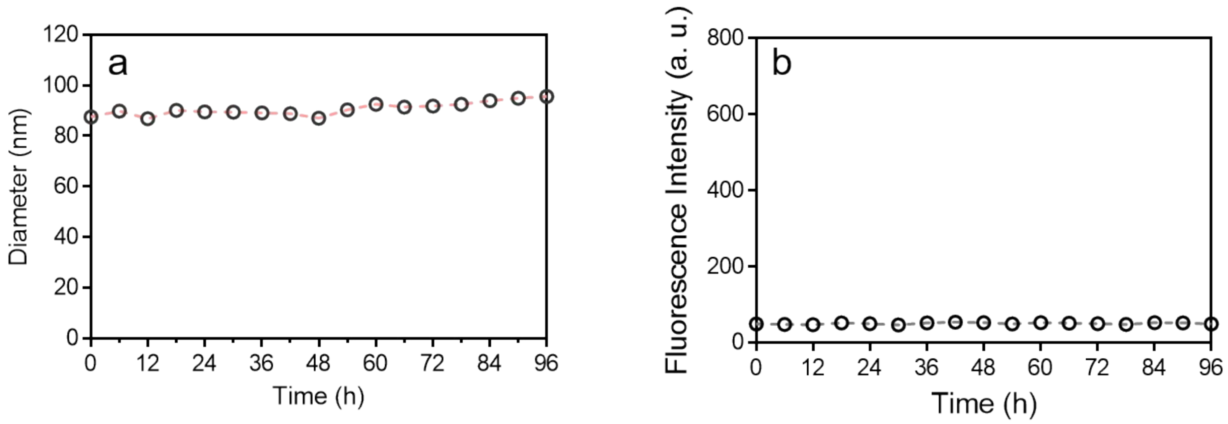

Fig. S2 (a) Time dependence of diameter of BP_{20}-DCM-S-CPT in blood serum over 4 days at 37 °C. (b) Fluorescence intensity at 650 nm of BP_{20}-DCM-S-CPT self-assemblies remain stable in fresh human serum over 4 days at 37 °C.

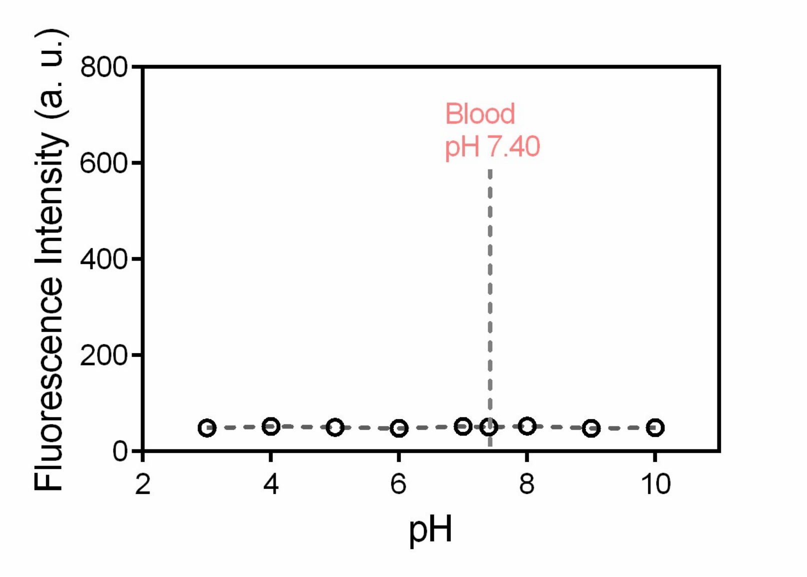

Fig. S3 Fluorescence intensity at 650 nm of BP_{20}-DC M-S-CPT self-assemblies as a function of pH value at 37 °C.

3. TEM Image of BP_5-DCM-S-CPT and BP_{20}-DCM-S-CPT & GSH

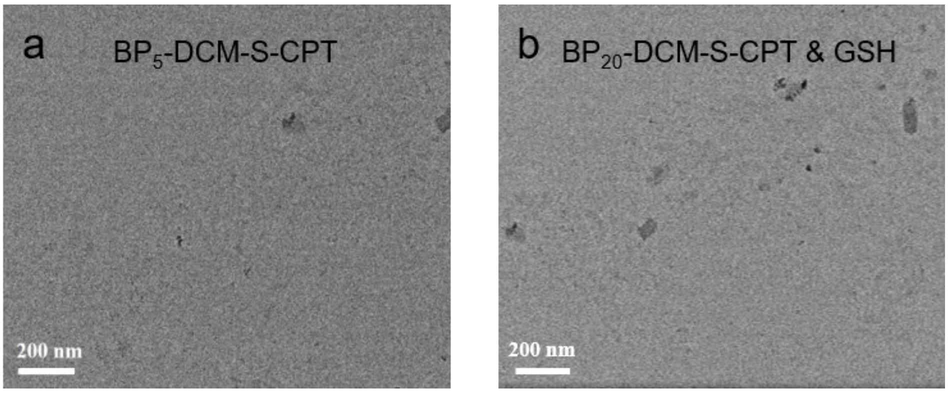

Fig. S4 (a) TEM image of BP_5-DCM-S-CPT at 10 μM in water. BP_5-DCM-S-CPT cannot form stable self-assemblies. (b) TEM image of BP20-DCM-S-CPT treated with GSH (2.5 mM GSH for 2 h).

4. Proposed Mechanism of BP$_n$-DCM-S-CPT Reaction with GSH

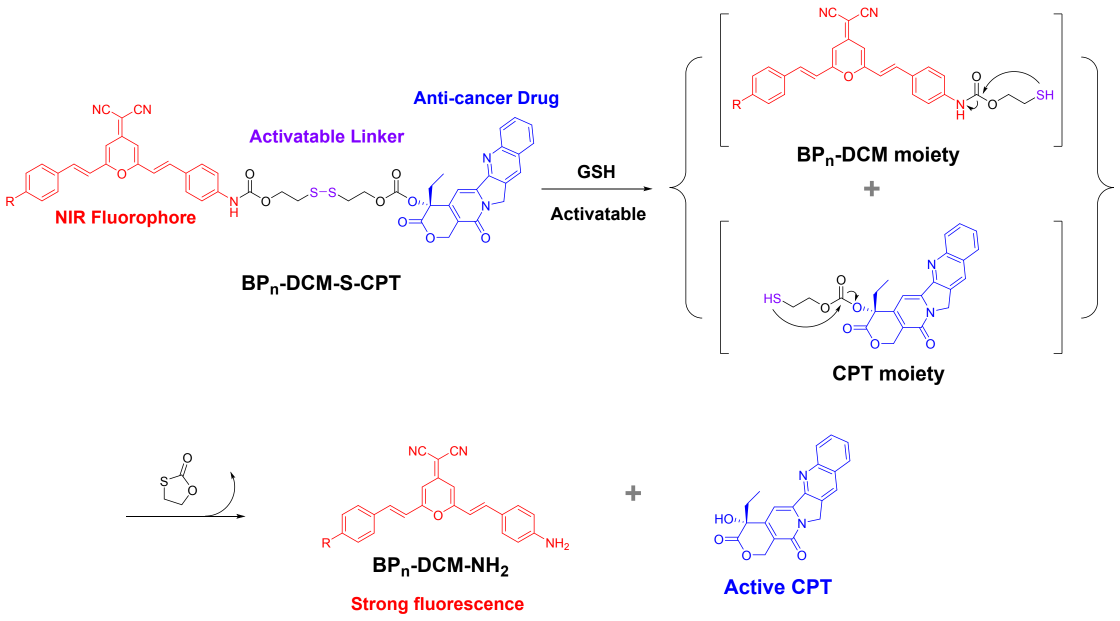

Scheme S2 Proposed mechanism in CPT activation and fluorescent variation of the prodrug BP$_n$-DCM-S-CPT by the treatment of GSH.

5. ESI-MS Spectra Characterization of DCM-S-CPT Reaction with GSH

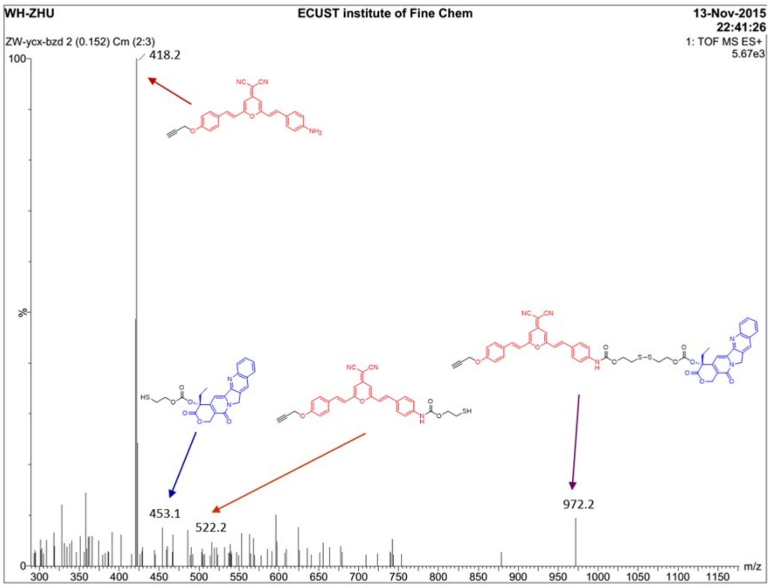

Fig. S5 HRMS spectrum of the products from the reaction of DCM-S-CPT with 20 equiv of GSH. Spectrum was obtained 1 h after exposure at 37°C.

6. Effect of Self-Assembled Nanostructure Formation on GSH-Induced Drug Release

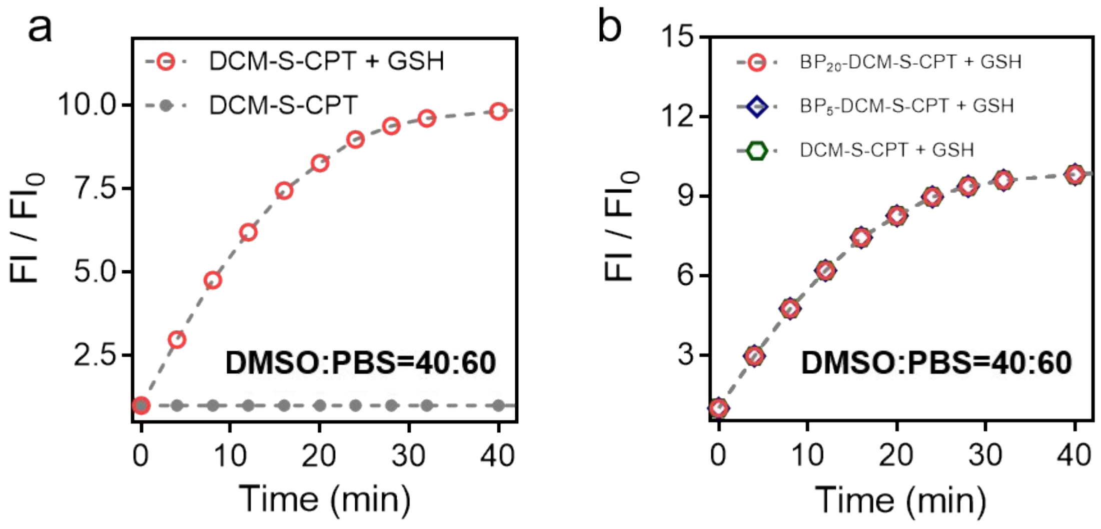

Fig. S6 (a) Time dependence of fluorescence intensity at 650 nm for DCM-S-CPT (10 μM) with and without GSH (2.5 mM) in DMSO/PBS buffer solution (40/60, v/v, pH = 7.4) at 37°C. (b) Time dependence of fluorescence intensity at 650 nm for DCM-S-CPT BP_5-DCM-S-CPT and BP_{20}-DCM-S-CPT (10 μM) with (2.5 mM) in DMSO/PBS buffer solution (40/60, v/v, pH = 7.4) at 37°C.

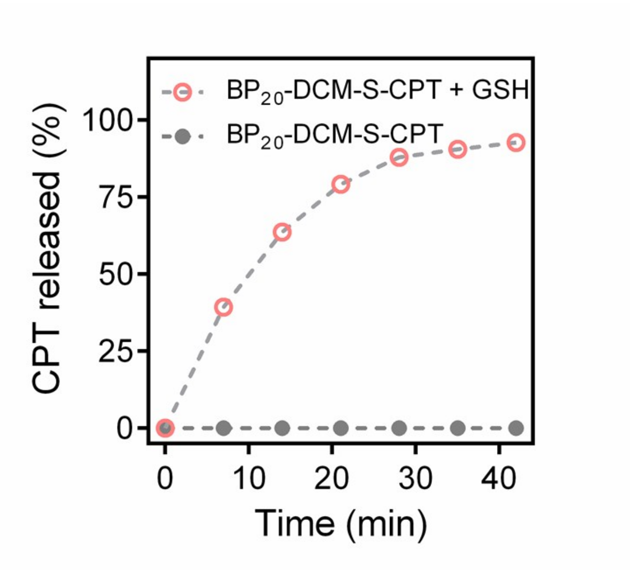

Fig. S7 CPT released from BP_{20}-DCM-S-CPT (in DMSO/PBS buffer solution, 40/60, v/v, pH = 7.4) as a function of time with and without GSH (2.5 mM) in HPLC chromatograms.

7. HPLC Chromatogram of BP_{20}-DCM-S-CPT

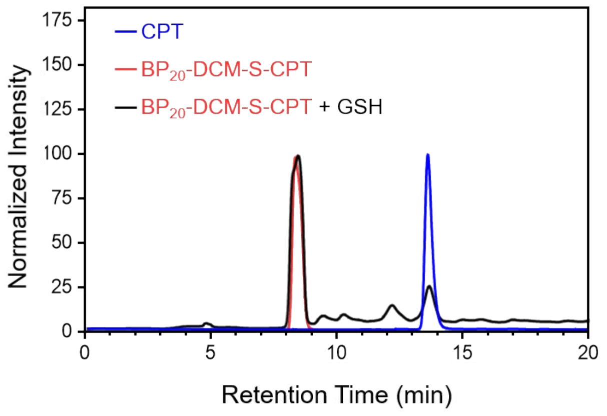

Fig. S8 HPLC analysis of GSH-driven drug release of BP_{20}-DCM-S-CPT at 10 min by monitoring the UV/Vis absorption at 254 nm. The retention time of BP_{20}-DCM-S-CPT and CPT is 8.19 min and 13.63 min respectively

8. Selectivity of BP_{20}-DCM-S-CPT

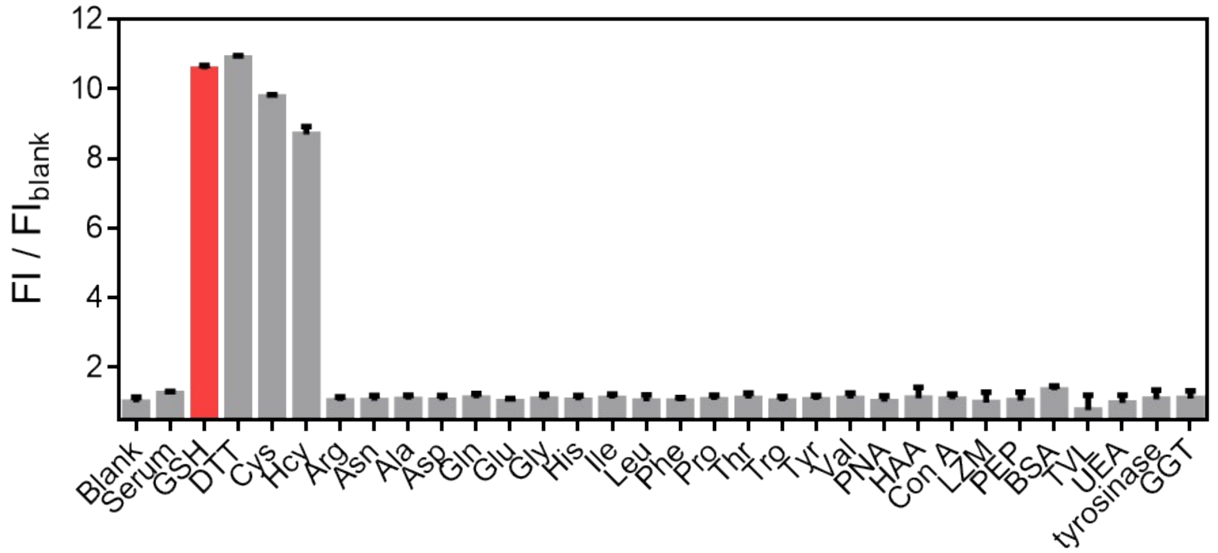

Fig. S9 Fluorescence responses (I_{650} nm) of BP_{20}-DCM-S-CPT toward various amino acids, enzymes and serum markers including GSH, DTT, Cys, Hcy, Arg, Asn, Ala, Asp, Gln, Glu, Gly, His, Ile, Leu, Phe, Pro, Thr, Tro, Tyr, Val, PNA (peanut agglutinin), HAA (snailagglutinin), Con A (concanavalin A), LZM (lysozyme), PEP (pepsin), BSA (bovine albumin), TVL (triticum vulgaris lectin), UEA (ulex europaeus lectin), tyrosinase, GGT (gamma-glutamyl transpeptidase)

9. Cytotoxicity of DCM-S-CPT, BP_5-DCM-S-CPT and BP_{20}-DCM-S-CPT

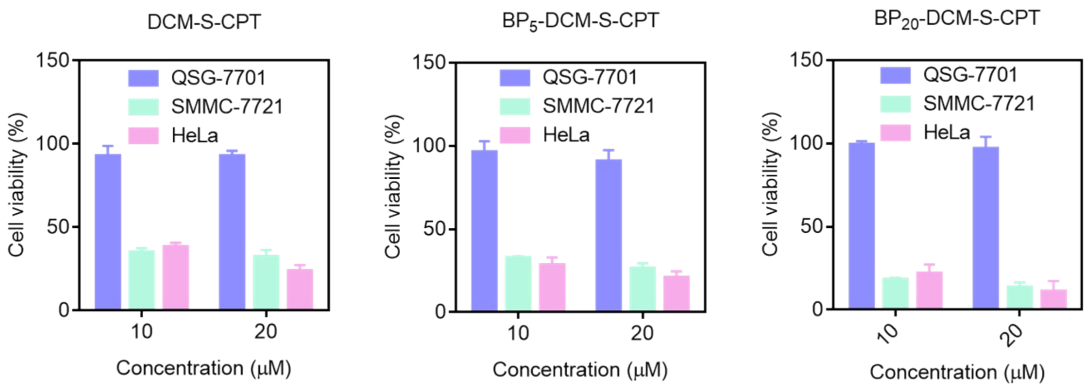

Fig. S10 *In vitro* dose-response relationship study of DCM-S-CPT (a), BP_5-DCM-S-CPT (b) and BP_{20}-DCM-S-CPT (c) on human hepatocyte QSG-7701 cells, human hepatoma SMMC-7721 cells and human epithelioid cervical carcinoma HeLa cells for 24 h.

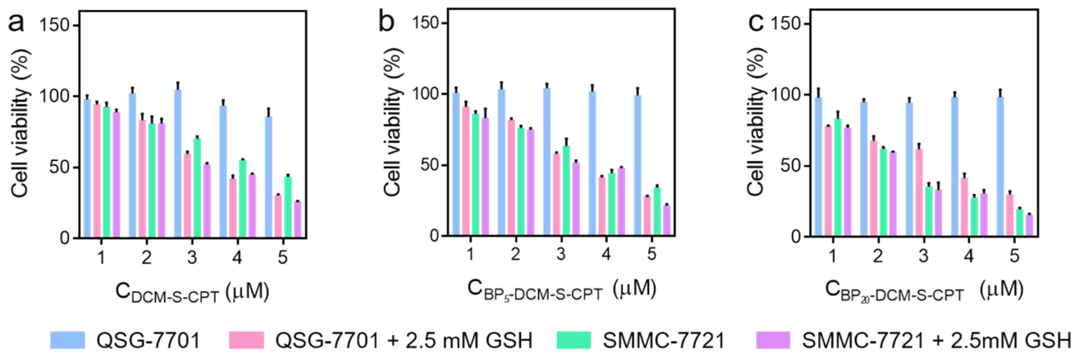

Fig. S11 *In vitro* dose-response relationship study of DCM-S-CPT (a), BP_5-DCM-S-CPT (b) and BP_{20}-DCM-S-CPT (c) with and without extra GSH (2.5 mM) on human hepatocyte QSG-7701 cells and human hepatoma SMMC-7721 cells for 24 h.

10. Confocal Laser Scanning Microscopy Images of HeLa Cells

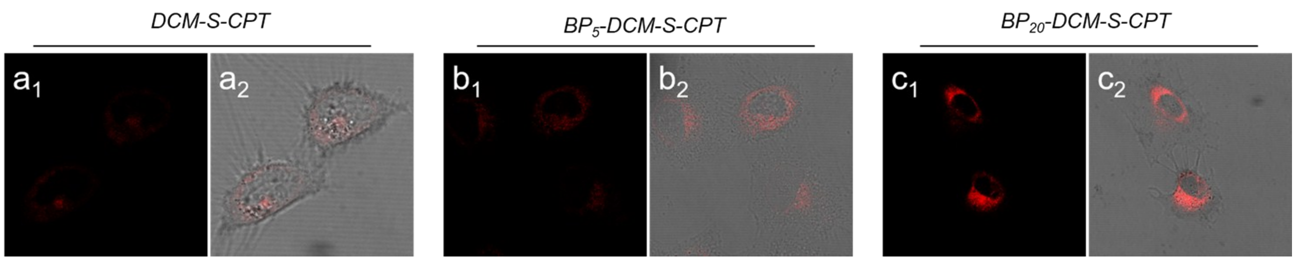

Fig. S12 Confocal laser scanning microscopy images (λ_{ex} = 488 nm, λ_{em} = 650-700 nm) of HeLa cells incubated with DCM-S-CPT (a), BP_5-DCM-S-CPT (b) and BP_{20}-DCM-S-CPT (c). Chanel 1: Fluorescence signal of activated prodrugs; Chanel 2: Overlapped field.

11. Antitumor Activity of DCM-S-CPT, BP_5-DCM-S-CPT and BP_{20}-DCM-S-CPT

Table S2 Changes of tumor volume of HeLa xenograft tumor-bearing mice after intravenous injection of PBS, CPT, DCM-S-CPT, BP_5-DCM-S-CPT and BP_{20}-DCM-S-CPT administered at a CPT-equivalent dose of 10 mg/kg every 3 days; each tumor was measured at the time of the injection and its relative tumor volume was calculated.

	No.	Tumor Volume (mm^3)						
		d0	d4	d7	d11	d14	d18	d21
PBS	1	87	148	411	690	893	1238	1558
	2	91	208	515	747	767	1337	1683
	3	104	239	403	522	628	1167	1363
	4	81	156	377	865	1201	1474	1880
	5	88	214	463	909	1368	1616	2166
	6	107	266	646	1013	1540	2129	2077
CPT	1	83	141	269	531	734	837	882
	2	87	138	264	459	619	700	760
	3	90	111	171	471	438	1041	1127
	4	99	126	190	266	370	470	454
	5	103	313	306	399	454	1283	1085
	6	127	161	306	540	690	1150	895
DCM-S-CPT	1	78	116	119	125	146	155	192
	2	86	127	216	241	257	293	467
	3	88	89	71	67	78	85	101
	4	101	130	167	140	256	192	266
	5	106	156	214	271	297	472	698
	6	124	140	130	167	157	199	265
BP_5-DCM-S-CPT	1	74	120	124	100	92	82	96
	2	85	79	85	81	66	55	54
	3	95	94	97	86	92	77	112
	4	100	119	167	152	123	75	53
	5	106	132	155	185	113	69	102
	6	125	162	153	151	110	107	96
BP_{20}-DCM-S-CPT	1	76	79	47	16	17	0	0
	2	88	78	44	24	20	0	0
	3	89	83	39	23	20	12	0
	4	101	77	61	31	20	11	0
	5	105	117	87	63	24	19	18
	6	114	130	98	63	51	26	0

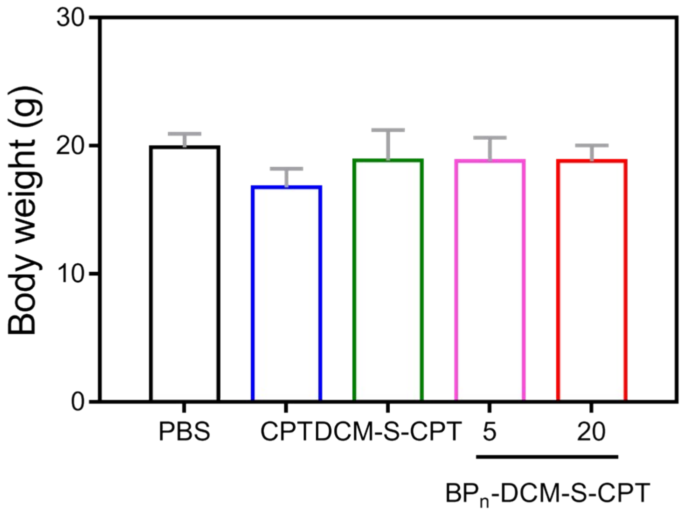

Fig. S13 The body weights of each group of the mice at the end of the experiment.

Table S3 Mean weight of tumors separated from mice after different treatments and inhibition rates of tumor growth (IRT) after treatment with CPT, DCM-S-CPT, BP_5-DCM-S-CPT and BP_{20}-DCM-S-CPT in HaLe tumor-bearing nude mice. The IRT is calculated using the following equation: IRT (%) = 100 × (mean tumor weight of control group - mean tumor weight of experimental group) / mean tumor weight of control group.

	Tumor Weight (g)						mean ± SD	IRT (%)
	1	2	3	4	5	6		
PBS	1.91	1.56	1.93	1.91	2.42	1.59	1.94 ± 0.31	
CPT	0.91	0.83	0.87	1.14	0.45	0.83	0.84 ± 0.22	55.57
DCM-S-CPT	0.08	0.28	0.31	0.26	0.56	0.24	0.29 ± 0.16	84.72
BP_5-DCM-S-CPT	0.13	0.07	0.07	0.15	0.15	0.09	0.11 ± 0.04	94.17
BP_{20}-DCM-S-CPT	0	0	0	0	0.04	0	0.01 ± 0.02	99.65

12. Characterization of Intermediate Compounds and DCM-S-CPT, BP_5-DCM-S-CPT and BP_{20}-DCM-S-CPT

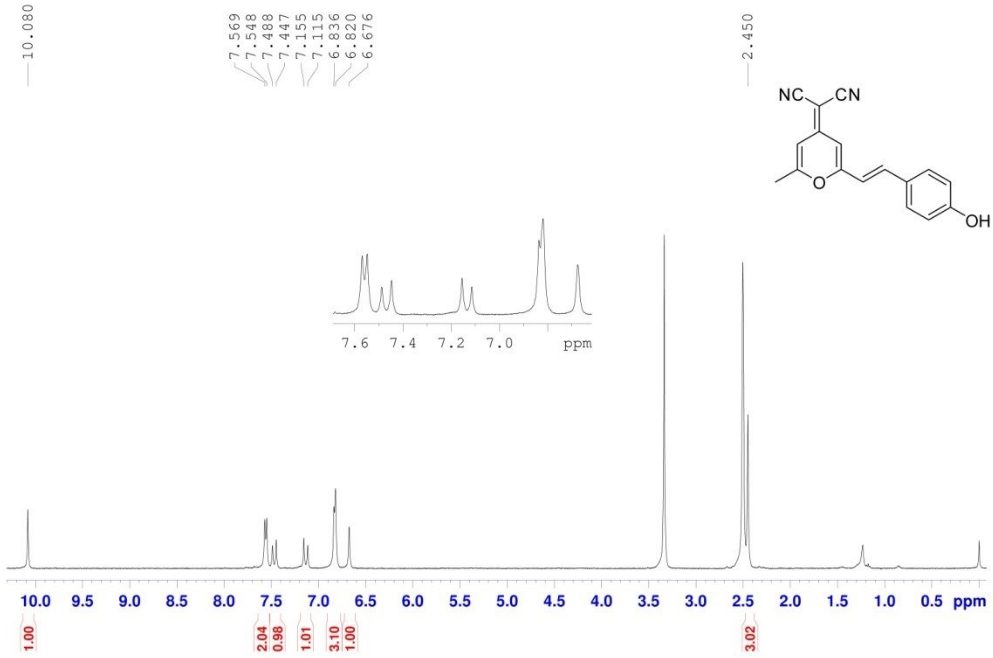

Fig. S14 ^{1}H NMR spectrum of DCM-OH in DMSO-d_6

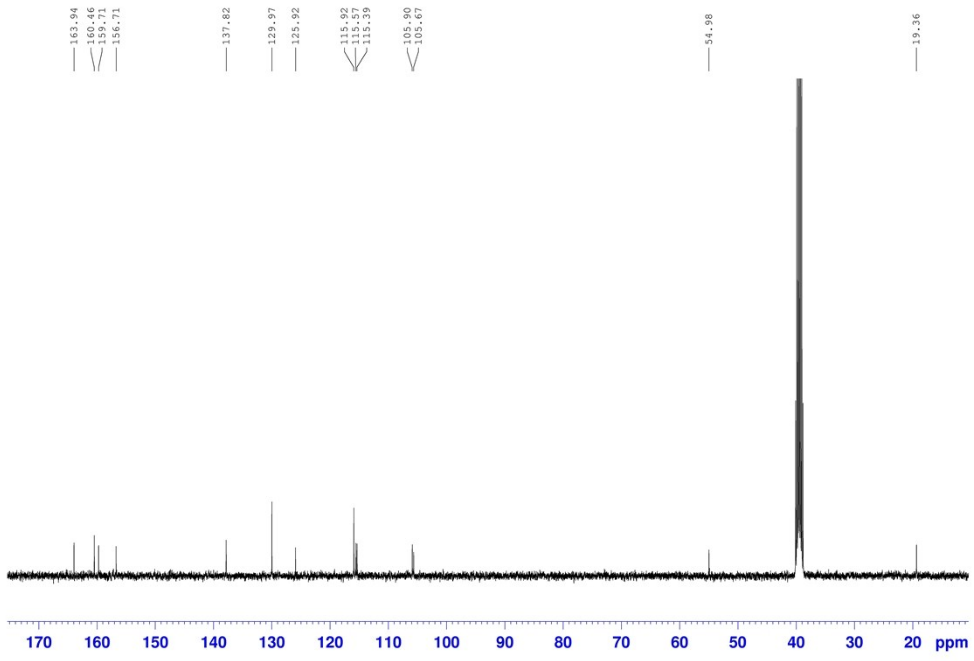

Fig. S15 ^{13}C NMR spectrum of DCM-OH in DMSO-d_6

Monoisotopic Mass, Even Electron Ions
125 formula(e) evaluated with 13 results within limits (up to 1 closest results for each mass)
Elements Used:
C: 0-25 H: 0-70 N: 0-4 O: 0-6

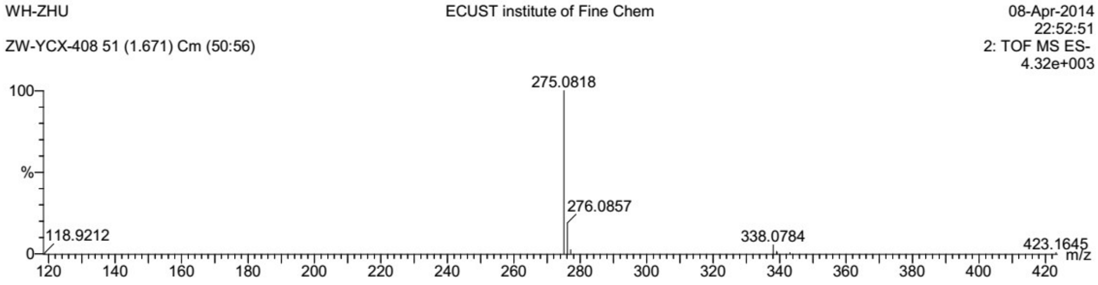

Minimum:				-1.5			
Maximum:		30.0	50.0	100.0			
Mass	Calc. Mass	mDa	PPM	DBE	i-FIT	i-FIT (Norm)	Formula
275.0818	275.0821	-0.3	-1.1	13.5	20.4	0.0	C17 H11 N2 O2

Fig. S16 HRMS spectrum of DCM-OH.

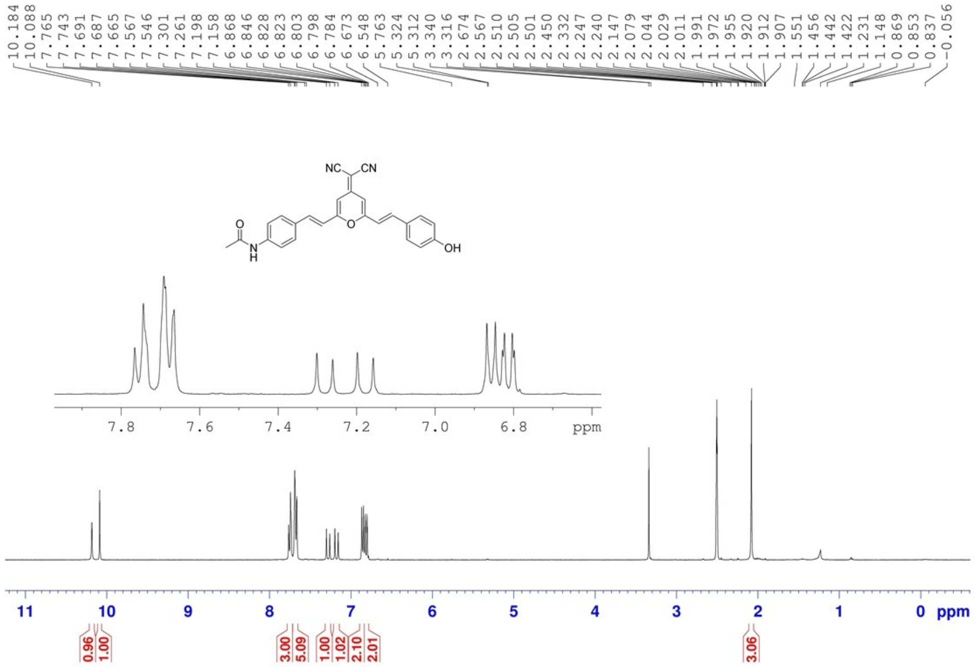

Fig. S17 ^{1}H NMR spectrum of BN-DCM-OH in DMSO-d_6

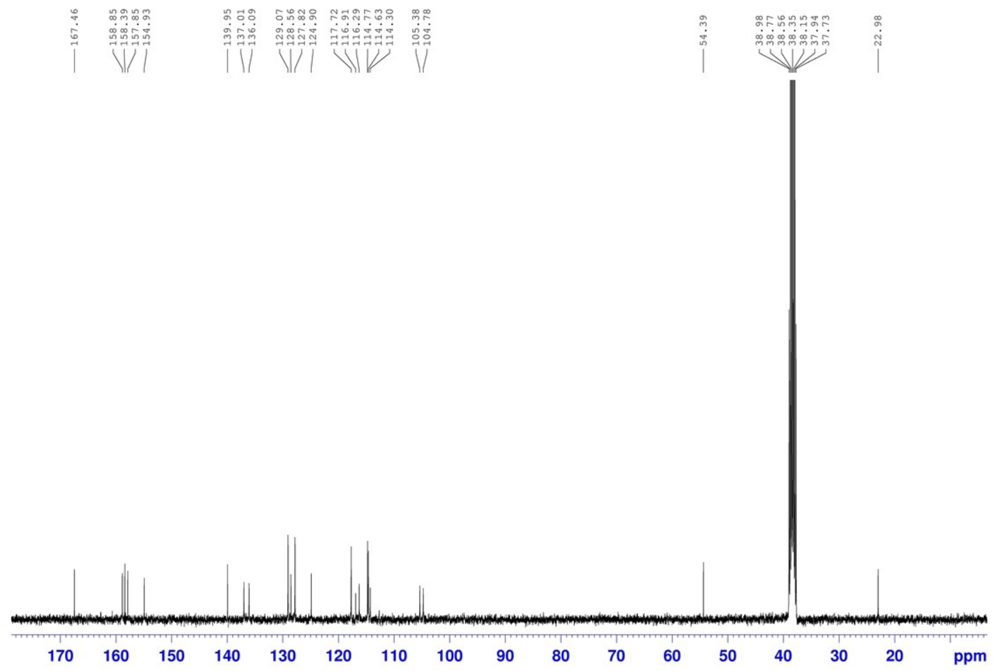

Fig. S18 ^{13}C NMR spectrum of BN-DCM-OH in DMSO-d_6

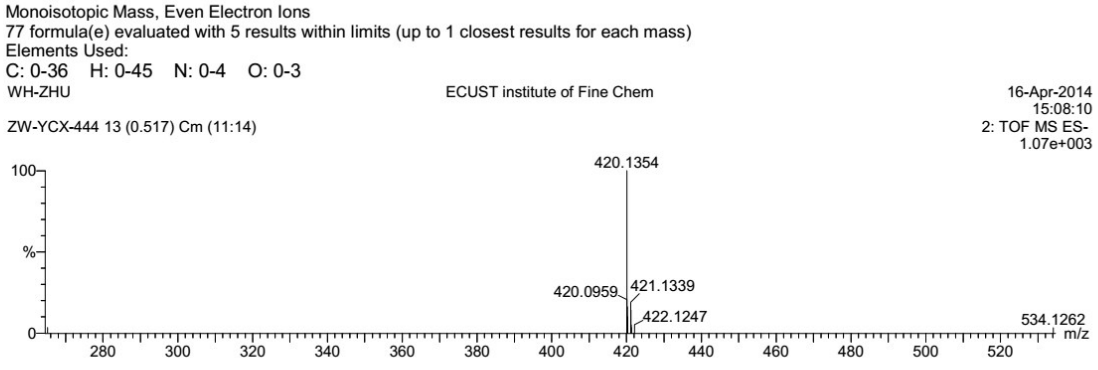

Minimum:				-1.5			
Maximum:		30.0	50.0	100.0			
Mass	Calc. Mass	mDa	PPM	DBE	i-FIT	i-FIT (Norm)	Formula
420.1354	420.1348	0.6	1.4	19.5	77.5	0.0	C26 H18 N3 O3

Fig. S19 HRMS spectrum of BN-DCM-OH.

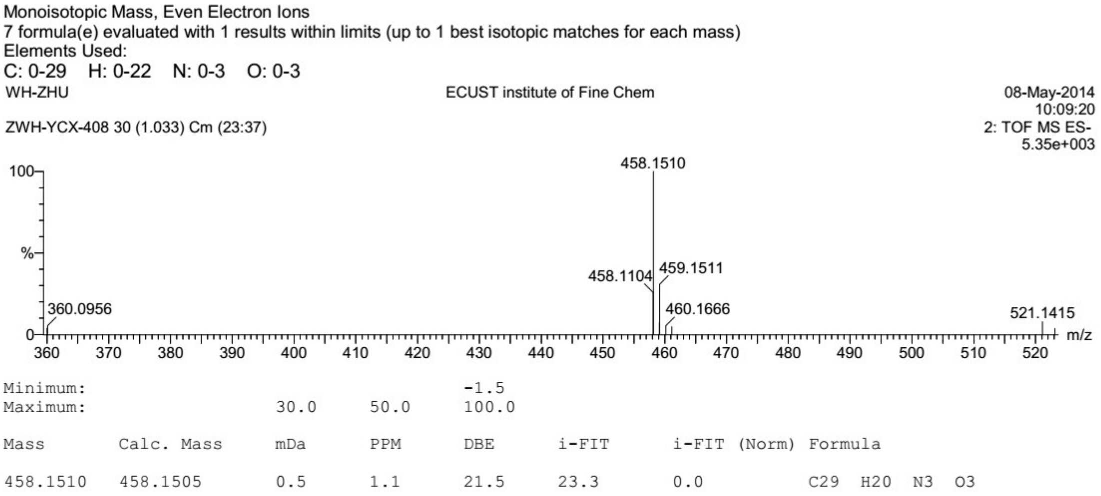

Fig. S20 HRMS spectrum of BN-DCM-A.

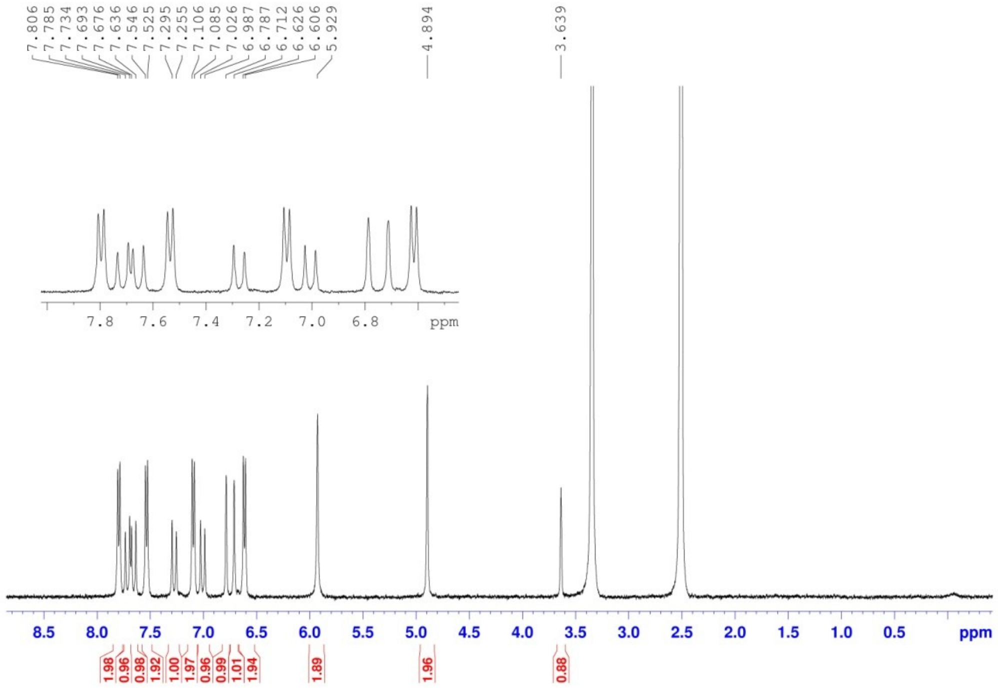

Fig. S21 ^{1}H NMR spectrum of A-DCM-NH_2 in DMSO-d_6

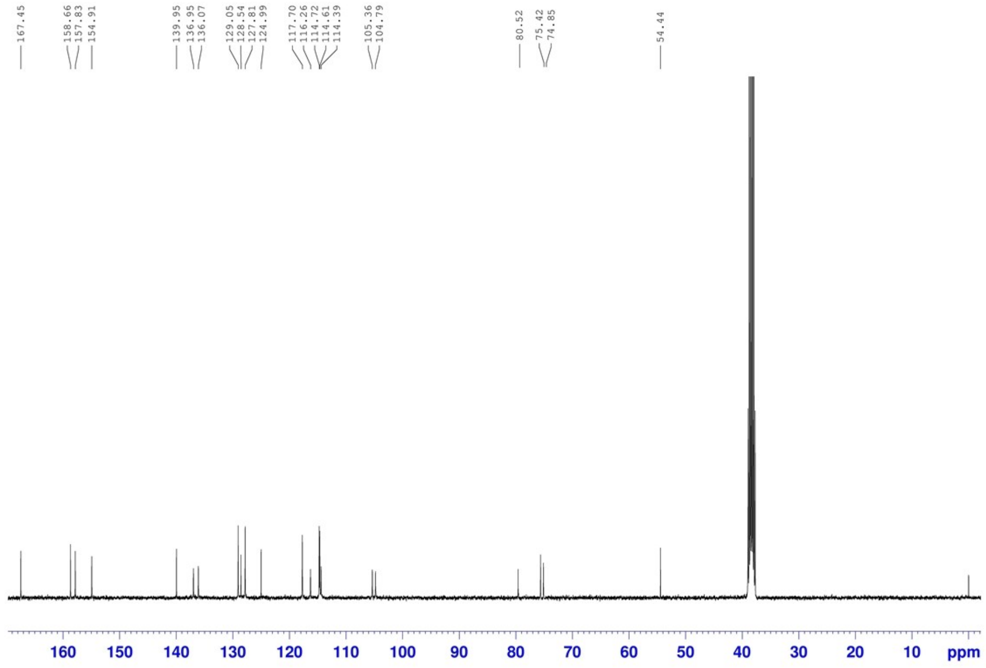

Fig. S22 ^{13}C NMR spectrum of A-DCM-NH_2 in DMSO-d_6

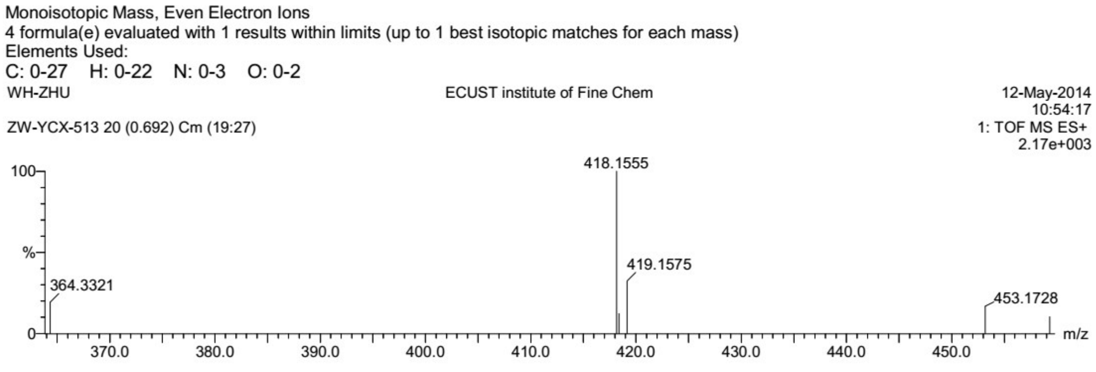

	Calc. Mass	mDa	PPM	DBE	i-FIT	i-FIT (Norm)	Formula
Minimum:				-1.5			
Maximum:		30.0	50.0	100.0			
Mass	Calc. Mass	mDa	PPM	DBE	i-FIT	i-FIT (Norm)	Formula
418.1555	418.1556	-0.1	-0.2	19.5	18.3	0.0	C27 H20 N3 O2

Fig. S23 HRMS spectrum of A-DCM-NH_2.

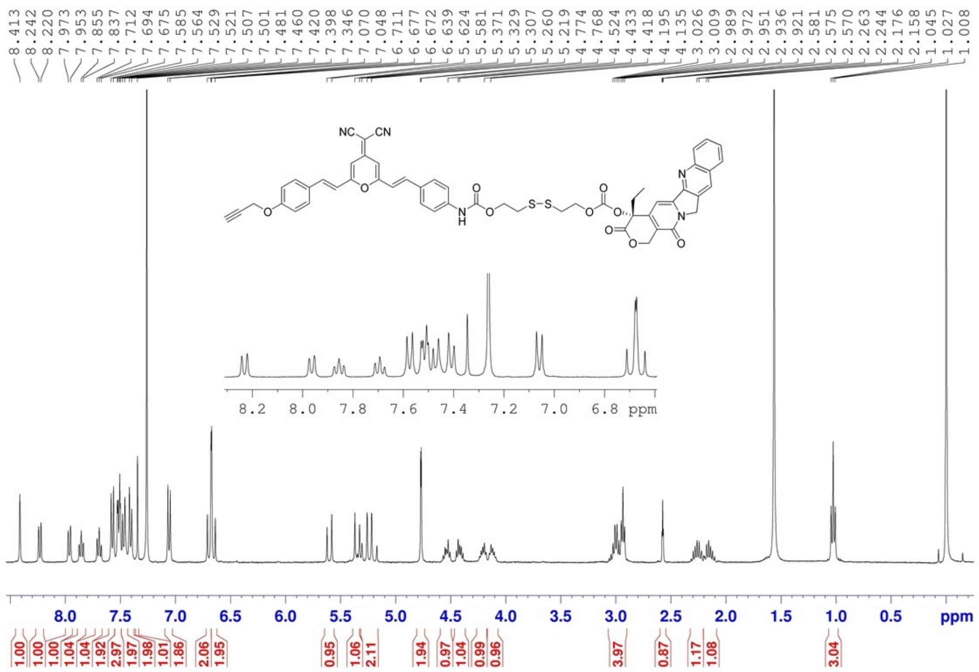

Fig. S24 ^{1}H NMR spectrum of DCM-S-CPT in $CDCl_3$

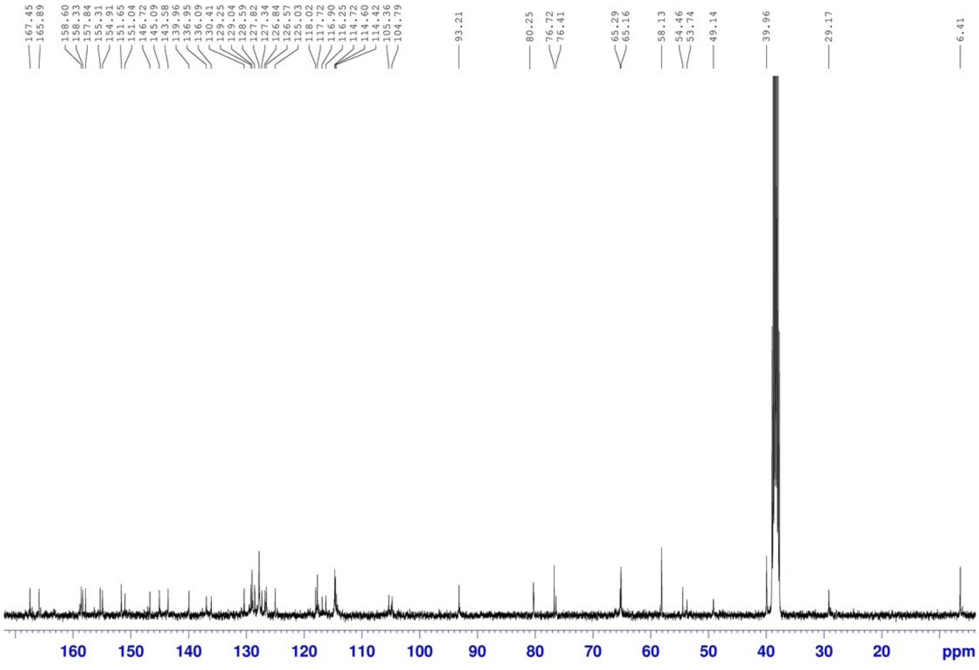

Fig. S25 ^{13}C NMR spectrum of DCM-S-CPT in DMSO-d_6

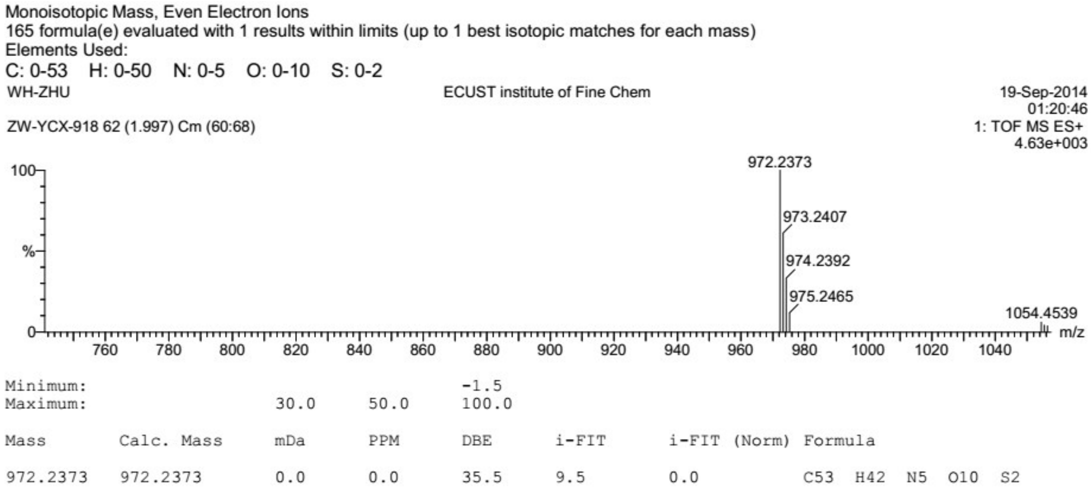

Fig. S26 HRMS spectrum of DCM-S-CPT.

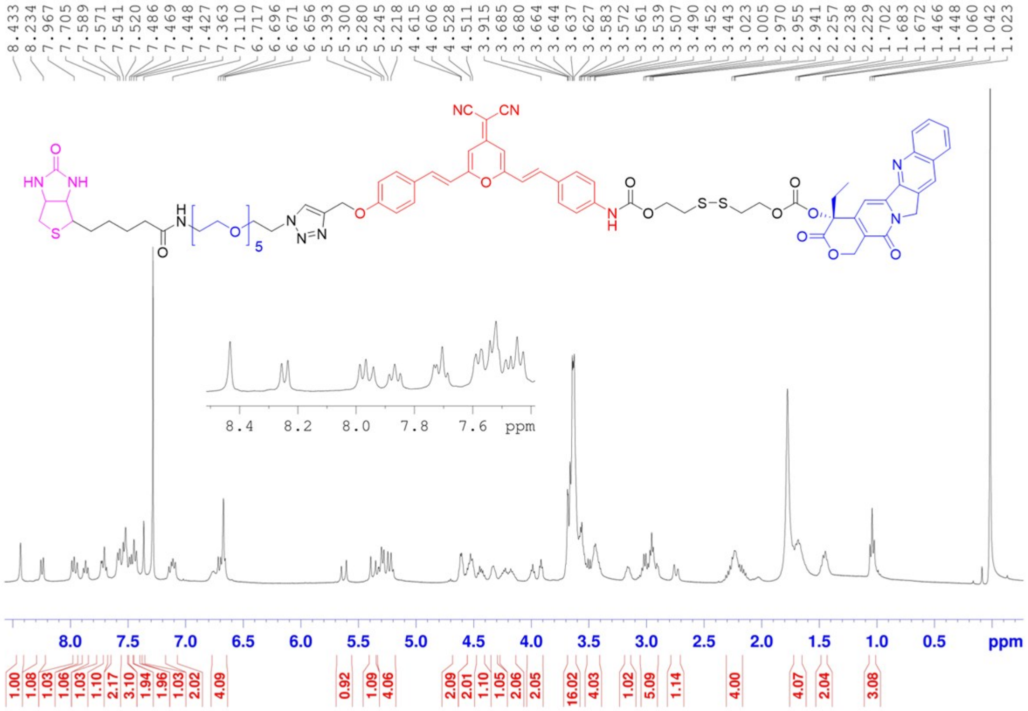

Fig. S27 ^{1}H NMR spectrum of BP_5-DCM-S-CPT in $CDCl_3$

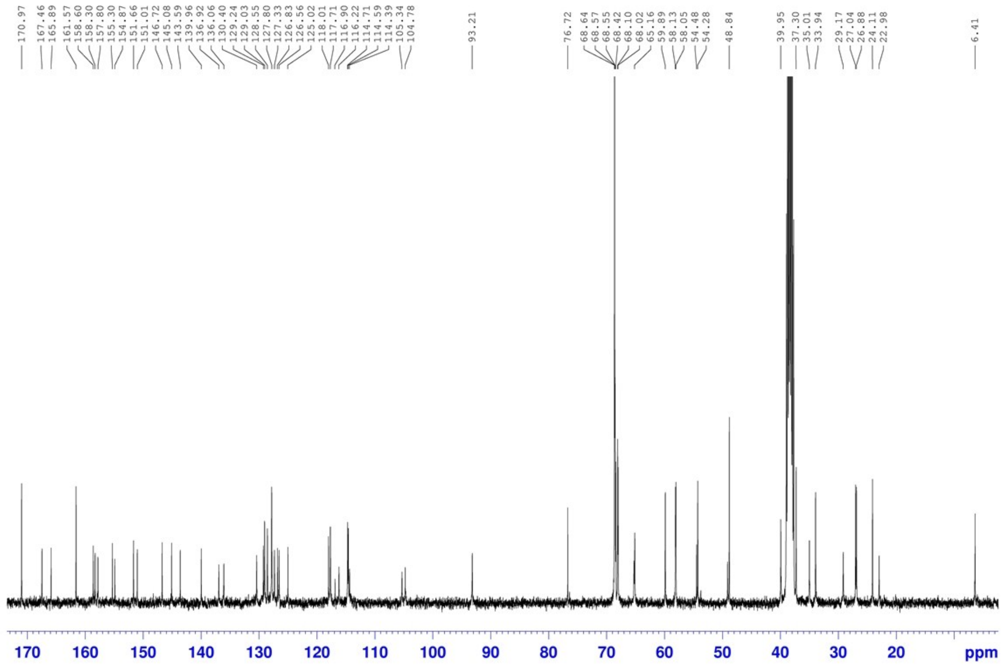

Fig. S28 ^{13}C NMR spectrum of BP_5-DCM-S-CPT in DMSO-d_6

Monoisotopic Mass, Even Electron Ions
829 formula(e) evaluated with 1 results within limits (up to 1 best isotopic matches for each mass)
Elements Used:
C: 0-75 H: 0-82 N: 0-11 O: 0-17 S: 0-3

ZL ECUST institute of Fine Chem 28-Apr-2015 17:14:21

ZL-CDD-04 43 (1.407) Cm (43) 1: TOF MS ES+ 2.20e+001

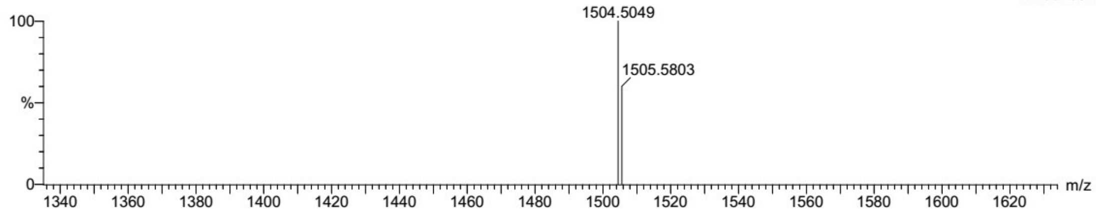

Minimum:				-1.5			
Maximum:		300.0	50.0	100.0			
Mass	Calc. Mass	mDa	PPM	DBE	i-FIT	i-FIT (Norm)	Formula
1504.5049	1504.5052	-0.3	-0.2	40.5	12.4	0.0	C75 H82 N11 O17 S3

Fig. S29 HRMS spectrum of BP_5-DCM-S-CPT.

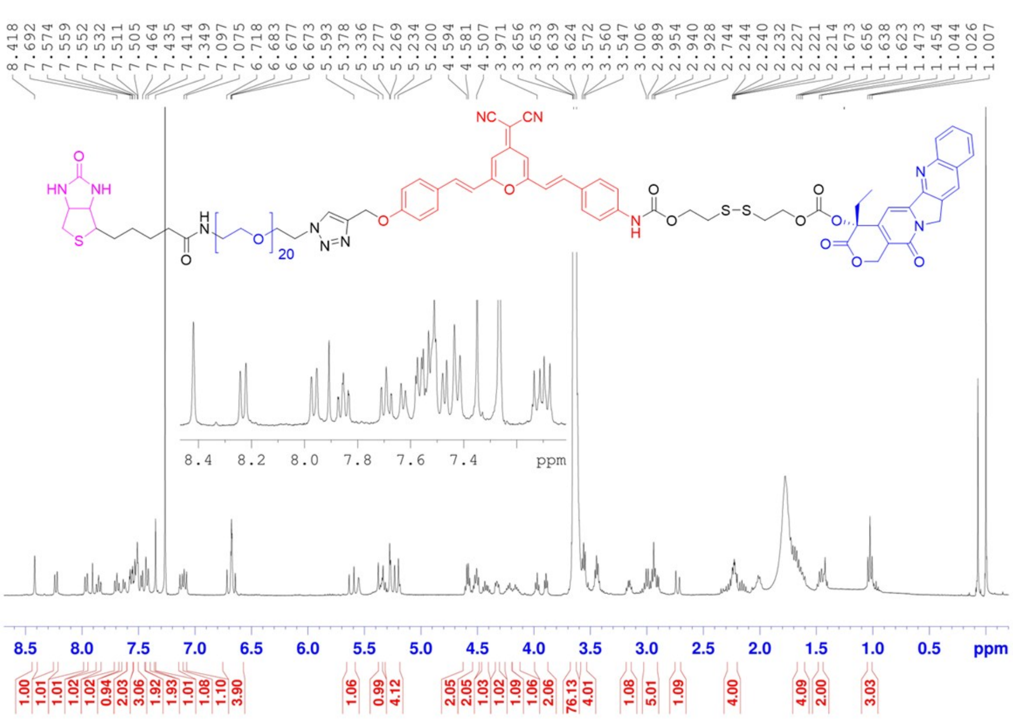

Fig. S30 ^{1}H NMR spectrum of BP_{20}-DCM-S-CPT in $CDCl_3$

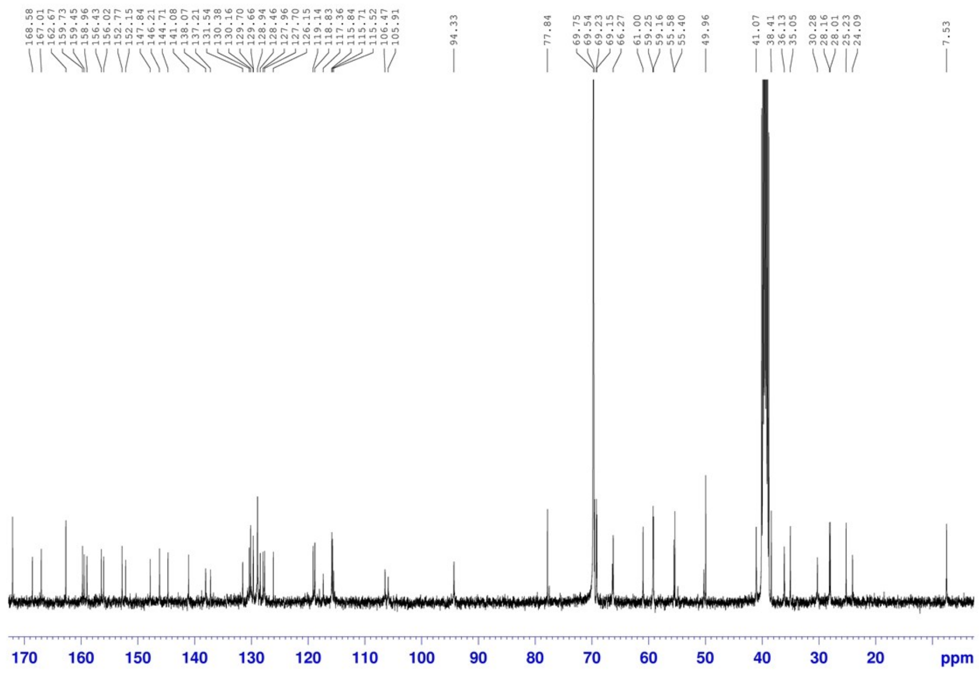

Fig. S31 ^{13}C NMR spectrum of BP_{20}-DCM-S-CPT in DMSO-d_6

Monoisotopic Mass, Even Electron Ions
1549 formula(e) evaluated with 1 results within limits (up to 1 best isotopic matches for each mass)
Elements Used:
C: 0-105 H: 0-142 N: 0-11 O: 0-32 S: 0-3

WH-ZHU ECUST institute of Fine Chem 10-Apr-2017 14:39:45
ZW-YCX-Z20 145 (1.871) Cm (145:151) 1: TOF MS ES+ 4.05e+000

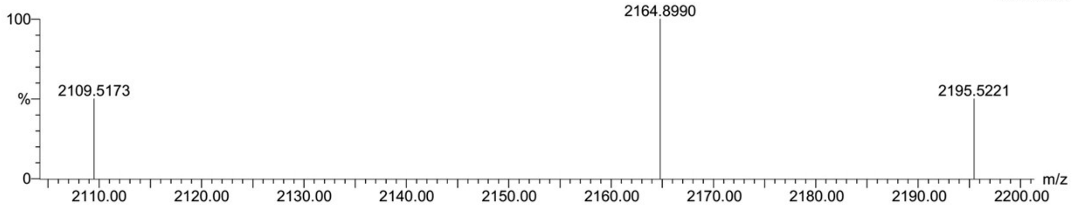

Minimum:				-1.5			
Maximum:		30.0	50.0	100.0			
Mass	Calc. Mass	mDa	PPM	DBE	i-FIT	i-FIT (Norm)	Formula
2164.8990	2164.8985	0.5	0.3	40.5	30.5	0.0	C105 H142 N11 O32 S3

Fig. S32 HRMS spectrum of BP_{20}-DCM-S-CPT.

References

1. M. Z. Ye, X. H. Wang, J. B. Tang, Z. Q. Guo, Y. Q. Shen, H. Tian and W. H. Zhu, *Chem. Sci.*, 2016, **7**, 4958-4965.
